# Primed to vocalize: Wild-derived male house mice increase vocalization rate and diversity after a previous encounter with a female

**DOI:** 10.1371/journal.pone.0242959

**Published:** 2020-12-09

**Authors:** Sarah M. Zala, Doris Nicolakis, Maria Adelaide Marconi, Anton Noll, Thomas Ruf, Peter Balazs, Dustin J. Penn

**Affiliations:** 1 Konrad Lorenz Institute of Ethology, University of Veterinary Medicine, Vienna, Austria; 2 Acoustic Research Institute, Austrian Academy of Sciences, Vienna, Austria; 3 Research Institute of Wildlife Ecology, University of Veterinary Medicine, Vienna, Austria; Liverpool John Moores University, UNITED KINGDOM

## Abstract

Males in a wide variety of taxa, including insects, birds and mammals, produce vocalizations to attract females. Male house mice emit ultrasonic vocalizations (USVs), especially during courtship and mating, which are surprising complex. It is often suggested that male mice vocalize at higher rates after interacting with a female, but the evidence is mixed depending upon the strain of mice. We conducted a study with wild-derived house mice (*Mus musculus musculus*) to test whether male courtship vocalizations (i.e., vocalizations emitted in a sexual context) are influenced by a prior direct interaction with a female, and if so, determine how long the effect lasts. We allowed sexually naïve males to directly interact with a female for five minutes (sexual priming), and then we recorded males’vocalizations either 1, 10, 20, or 30 days later when presented with an unfamiliar female (separated by a perforated partition) and female scent. We automatically detected USVs and processed recordings using the Automatic Mouse Ultrasound Detector (A-MUD version 3.2), and we describe our improved version of this tool and tests of its performance. We measured vocalization rate and spectro-temporal features and we manually classified USVs into 15 types to investigate priming effects on vocal repertoire diversity and composition. After sexual priming, males emitted nearly three times as many USVs, they had a larger repertoire diversity, and their vocalizations had different spectro-temporal features (USV length, slope and variability in USV frequency) compared to unprimed controls. Unprimed control males had the most distinctive repertoire composition compared to the primed groups. Most of the effects were found when comparing unprimed to all primed males (treatment models), irrespective of the time since priming. Timepoint models showed that USV length increased 1 day after priming, that repertoire diversity increased 1 and 20 days after priming, and that the variability of USV frequencies was lower 20 and 30 days after priming. Our results show that wild-derived male mice increased the number and diversity of courtship vocalizations if they previously interacted with a female. Thus, the USVs of house mice are not only context-dependent, they depend upon previous social experience and perhaps the contexts of these experiences. The effect of sexual priming on male courtship vocalizations is likely mediated by neuro-endocrine-mechanisms, which may function to advertise males’ sexual arousal and facilitate social recognition.

## Introduction

Males in many species produce complex courtship vocalizations to attract females, which can provide information about their quality and compatibility to potential mates [1–4]. In some taxa, such as insects, amphibians, rodents, and bats, individuals communicate through ultrasonic vocalizations (USVs) (>20 kHz) [5–8]. Male house mice (*Mus musculus)* produce surprisingly complex USVs, which show features of bird song [9] (reviewed in [10]). Males mainly emit USVs upon encountering females or their scent [11–14], and their vocalizations become more complex during courtship and mating [15]. Vocalizations emitted by male mice during sexual contexts are widely referred to as *courtship ultrasonic vocalizations* (cUSVs) [8, 12, 16, 17], though these vocalizations may have other functions. Both sexes vocalize [18], but males emit most of the USVs during direct opposite-sex interactions [19]. Males vocalize at high rates during anogenital sniffing and copulation [13, 16, 20–22], whereas they cease vocalizing abruptly after ejaculation [13, 20, 23, 24]. Females are attracted to playbacks of male USVs [14, 25–27], and these vocalizations can enhance mating and reproductive success [17, 28, 29]. Females are more attracted to vocalizations of males of their own versus other *Mus* species [30] and to the USVs of unrelated males over siblings [14]. The USVs of house mice are innate in the sense that they do not require vocal learning [31]. Nevertheless, the number and types of vocalizations that male mice produce depends upon their internal state and social and sexual contexts [32], which may be influenced by previous experience and perception of potential mating opportunities. USVs may provide indices of an individual’s emotional state [33], and may signal a male’s sexual arousal and interest in a potential mate.

Numerous studies on laboratory mice have suggested that male cUSVs are influenced by previous socio-sexual experience, and a variety of different approaches have been used to investigate this hypothesis, from a brief exposure to mouse scent to direct long-term interactions and copulation. Several early studies reported that the rate of male cUSV emission is increased after a previous encounter with a female or female scent, and that even a brief experience may have long-lasting effects (persisting >1d) [reviewed in 12, 34–39]. These early findings are intriguing, and they also suggested that male cUSVs provide a reliable index of sexual arousal. However, the results were mixed and varied depending on the strain of mice, the sex and type of stimuli (direct social interactions vs urine odour, and fresh vs aged urine), and only vocalizations at 70 kHz were recorded due to technical limitations. One previous study concluded that socio-sexual experience is only necessary to elicit male USVs when males are presented with aged female urine as a stimulus [38]. Since then, it has been anecdotally suggested that exposing male mice to a sexually mature female for several days or more before recording increases their motivational state to emit cUSVs [40, 41], but this hypothesis was not tested. Another recent study exposed individual mice (strain CBA/CaJ) to another mouse indirectly (separated with a metal mesh divider) for one hour, and then vocalizations were immediately recorded in a novel environment and without any stimulus (non-sociosexual context) [42]. Males (but not females) showed increased USV emission after an experience with females (or even males) compared to isolated stimulus-deprived controls, which had no prior social exposure. This result shows that males increased their number of *spontaneous* USVs directly following a socio-sexual experience. An additional study recorded USVs of laboratory mice (strain C57BL/6J) during direct male-female interactions [17]. The study found increased rates of USV emission among pairs if the male had been previously co-housed with a female for two weeks, whereas pairs with individually housed males did not show such changes. The increased number in USVs might have been due to socio-sexual experience (either prior copulation or long-term co-housing with a female), female vocalizations (pairs where recorded while directly interacting), or both. Thus, studies are still needed to determine whether a previous direct interaction with a female is sufficient to influence the number and types of male vocalizations in a sexual context, and if so, then how long such effects last.

These previous studies were conducted with laboratory strains (*Mus laboratorius*), and it is unclear whether the findings generalize to wild house mice or to other strains. Laboratory mice have been artificially selected for rapid breeding, and males quickly initiate courtship and mating behaviour upon perceiving another mouse, even another male (see Discussion). Wild-derived males vocalize during interactions with females or their scent [14] and both sexes vocalize at a higher rate during opposite- compared to same-sex interactions [18]. They show enormous inter-individual variation in USV emission [18]; however, unlike laboratory mice, wild-derived male mice vocalize very little, if at all, when they are alone [43], and require direct contact with another mouse or mouse scent [14]. Only one study to our knowledge has investigated socio-sexual experience and USV emission in wild-derived house mice. Males were exposed to a stimulus mouse (separated by a perforated divider) and then recorded at least 5 days later in a sexual context (presentation of female odour) [14]. The number of male cUSVs emitted were not altered by previous exposure to a male or female conspecific; however, the socio-sexual experience regime was limited in this study and it may not have been sufficient for priming, as the authors acknowledged.

We conducted a study on the courtship vocalizations of wild-derived male mice *(Mus musculus musculus)* and our aims were to test whether previous exposure to a female mouse in direct but brief interactions (sexual priming) influences the number of courtship vocalizations (sonic and ultrasonic), the types of USVs, and the spectro-temporal features of USVs compared to control males not previously exposed to a female. To address how long the effects of sexual priming last, we compared a null model, a treatment model (controls vs all primed males, irrespective of the time since priming) and a timepoints model including all 5 groups: before priming and day 1, day 10, day 20 and day 30 after priming. We introduced a female into a male’s cage and allowed the mice to interact for 5 min (sexual priming). The mice never copulated during this time. We subsequently recorded the male courtship vocalizations either 1, 10, 20 or 30 d after sexual priming. We predicted that sexual priming would increase male USVs emission compared to unexposed controls, and that this effect might decline over time. We detected USVs and processed the recordings using an improved version of the Automatic Mouse Ultrasound Detector (A-MUD) [44]. We describe the improvements of A-MUD (version 3.2), which enable users to adjust the detection threshold and to automatically assign a quality evaluation score to each putative vocalization detected (i.e., A-MUD elements), and we provide the results of our evaluation of A-MUD’s performance. Sexual experience might influence the types of different USVs and their diversity, as well as the rate of vocalizations that males emit. Therefore, we also manually classified USVs into 15 different vocalization types ('syllables'), and investigated changes in vocal repertoire (repertoire diversity and composition of USVs). These questions are relevant to understanding the proximate mechanisms and adaptive functions of male USV emission, and they are also of practical interest, as sexual priming has become a common procedure for eliciting vocalizations from male mice [45].

## Materials and methods

### Subjects and housing

Our experiment was conducted with wild-derived house mice (*Mus musculus musculus*), which were F1 offspring of wild house mice caught and bred at the Konrad Lorenz Institute of Ethology in Vienna, Austria (48°12’38”N, 16°16’54”E) [see more details in 18]. We systematically bred mice that we trapped from different locations and made crosses among them (mean±s.d. distance between locations 85 ± 71 m). The mice were housed in standard Type IIL cages (36.5 x 20 x 14 cm, with stainless steel cover, 1 cm mesh width, Tecniplast, Germany). The F1 offspring were weaned at 21 d of age and subsequently housed with their siblings in mixed-sex groups for another two weeks (maximum of four mice per cage). At five weeks of age, the sexes were separated. Males were individually housed to prevent fighting and females were housed in sister pairs when possible. All cages were provided with wood shavings (ABEDD, Austria), one nest box (Tecniplast, Germany), nesting material (Nestlet, Ehret, Austria) and one cardboard paper roll as environmental enrichment. Food (rodent diet 1324, Altromin, Germany) and water were provided *ad libitum*. Mice were kept in standard conditions (mean±s.d. room temperate: 22±2°C, in a 12:12 h light:dark cycle, lights off at 15:00). Red light was used instead of a complete dark period to be able to conduct experiments when the mice are active. We used 100 adult mice (equal sex ratio; mean±s.d. age: 264±22 d).

### Recording apparatus

To record male vocalizations, we used a Plexiglas cage (36.5 x 21 x 15 cm), which had a perforated Plexiglas divider (0.5 cm diameter holes) in the middle to create two equal sides (“caller” and “stimulus” compartments; for details see [18]). The caller compartment (used for the males) had a metal cage lid (1 cm width mesh), and the stimulus compartment (for the females) was covered with a Plexiglas lid, which prevented recording female vocalizations during the experiment. We used USV playbacks from an ultrasound speaker (Avisoft Bioacoustics, Germany) positioned into the stimulus compartment to confirm that the Plexiglass cover was very effective at blocking USVs [18]. The stimulus compartment was also provided with bedding and 2–3 food pellets. For recording, the Plexiglas cage was placed into a recording chamber, lined with acoustic foam, as described in [30]. A condenser ultrasound microphone (Avisoft Bioacoustics/CM16/CMPA, frequency range from 2 to 200 kHz) was mounted inside the recording chamber, 10 cm above the caller compartment, and connected to an UltraSoundGate 116–200 (Avisoft Bioacoustics, Germany). Mice were recorded using the RECORDER USGH-software and with the following settings: 300 kHz sampling rate, 16 bit format and 256 Hz FFT size.

### Socio-sexual priming and recording procedures

For our priming treatment, we introduced an unfamiliar adult female (n = 40) into a male’s home cage (n = 40) for 5 min. Wild mice never copulate during such a brief period of time—unlike laboratory mice, it usually takes wild-derived mice days to copulate [46], rather than minutes or hours. We subsequently recorded 10 of these males 1 day later in a sexual context, while presenting them with a novel stimulus female (separated by a perforated partition) and female scent (see below). To investigate whether priming effects are long-lasting, the rest of the males were recorded either 10, 20 or 30 d after priming, using 10 males per time point. We also recorded a control group composed of 10 unprimed males. In total we compared five groups: unprimed males (0d), males primed 1 d prior to recordings (1d), males primed 10 d before recordings (10d), males primed 20 d prior to recordings (20d) and males primed 30 d before recordings (30d). Each group contained 10 males and no male was recorded on more than one day. To prevent the data from being confounded by time and sequence effects during testing, we primed and recorded the males during two months by priming and respectively recording the males in the following order: 1d, 30d, 20d, 10d (priming), and 1d, 0d 10d, 20d and 30d (recording).

We recorded males’ vocalizations using the following procedure: first, a female stimulus was placed into the stimulus compartment of the arena for habituation, 5–10 min before introducing the male. To standardize any potential oestrus status effects of this stimulus female, we included an additional olfactory stimulus (5 μl of female urine on a 4 x 4 cm filter paper) to the male compartment. This urine stimulus was previously collected in metabolic cages (Techniplast, 600M021) from four wild-caught adult females, equally aliquoted and mixed in Eppendorf tubes and stored at -20°C until the recordings. Second, we placed the male subject into the caller compartment and the entire cage was placed into the recording chamber. After 30 s, we began recording and we recorded males for 10 min. After each recording, the arena was cleaned with ethanol before reusing. Each male subject was unfamiliar and unrelated to the females that he encountered in the experiment. Females were used once for priming and once in the stimulus compartment, but never for the same male subject. This experiment was part of a larger study aimed to test whether USV modulation is sex-dependent [18], and 10 males in the current study (i.e., males tested 1 day after sexual priming) were the same as the ‘male focal subjects presented with female stimuli’ in our previous study.

### Detecting vocalizations and processing sound files

To detect ultrasonic vocalizations and process the sound files, we implemented the Automatic Mouse Ultrasound Detector (A-MUD), which detects elements (i.e., putative vocalizations detected by A-MUD) and quantifies spectro-temporal features such as the frequency, amplitude and time parameters of the elements [18, 44]. This tool is implemented as a script in STx (requiring at least S_TOOLS-STx version 4.3), a software from the Acoustic Research Institute (Austria) that is free for scientific use, and is useful for processing large quantities of data in a timely fashion, such as for speech analysis [47, 48], noise evaluation [49, 50], and psychoacoustics [51]. We developed an improved version of A-MUD (version 3.2) and evaluated its performance (see S1 Methods in S1 File). In brief, A-MUD 1.0 has a *detection threshold* at 10 ms because sounds below this threshold are often background noise, and this threshold can now be adjusted by users. For the present study, we lowered the detection threshold to 5 ms to reduce false negative error rates in element detection, despite that this modification increases the risk of false positives. This trade-off was acceptable for the current study because, after the automatic detection, we manually classified all vocalizations in each 10 min file (see below), and thus we were able to correct the output as necessary. A-MUD 3.2 also includes a *quality evaluation score* for each detected A-MUD element, which provides an estimate in the confidence of a true positive, and enables users to remove segments below a certain criterion from the data (the score varies from 0 to 9 with segments ≥ 5 being of good quality). A well-established method to judge the performance of a detection or classification model is the receiver operating characteristic (ROC) curve, which plots the true positive rate (TPR) against the false positive rate (FPR). The area under the ROC curve (AUC) is one value for the quality of the method (see S1 File for details). We used a ROC curve to evaluate A-MUD’s performance using the default settings and the original 14 files used to develop and evaluate A-MUD 1.0 [44], and the AUC value was 0.989 (values > 0.9 are considered to be excellent; see S1 to S4 Figs in S1 File).

**Fig 1:**
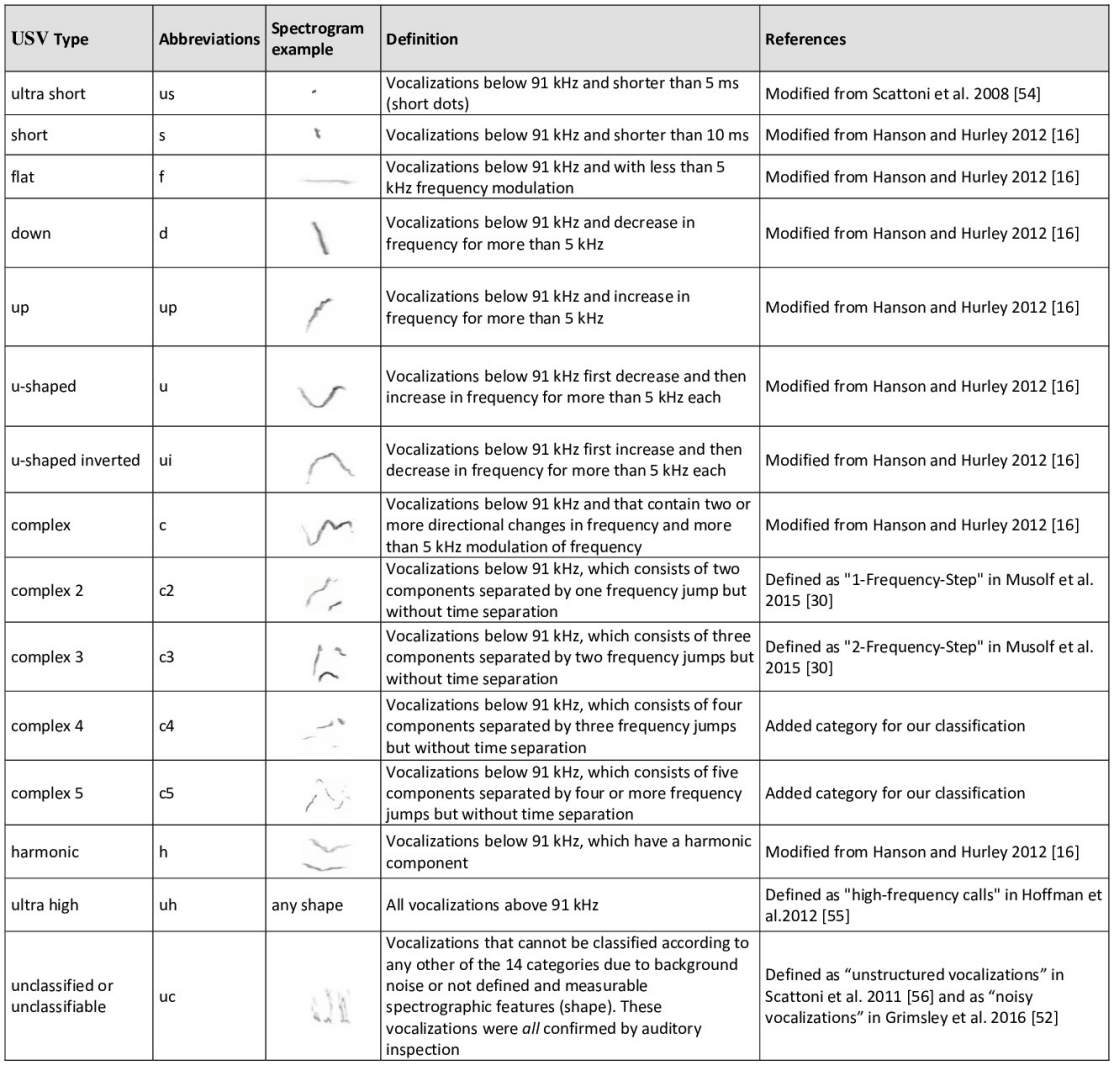
Classification of USVs: USV types, their abbreviations, a spectrogram’s example and definitions following the classification of [28, 54–56].

After automatic detection, we manually classified each ultrasonic vocalization, assigning it to one of the 15 different previously described types (see Fig 1). We classified and analysed all vocalizations in all 10 min files. This work is very time-consuming, but it allowed us to test for priming effects in the ultrasonic vocal repertoire (ultrasonic repertoire diversity and composition, see below), as well as to correct errors of the automatic detection (eliminate false positives, or manually label false negatives and adjust the vocalization length) and then recalculate the USV spectro-temporal parameters in A-MUD. Manual classification also allowed us to additionaly identify and count *sonic vocalizations* (all vocalizations < 20 kHz). We classified sonic vocalizations into two main categories: *low-frequency vocalizations* (LFV), which are similar to USVs but are at frequencies < 20 kHz (adapted from [52]) and *low-frequency harmonic vocalizations* or *squeaks*, which are qualitatively distinct and are vocalizations showing > 1 harmonic component, starting at the sonic range and often reaching the ultrasonic range [32, 52]. The rationale was to test whether the number of ultrasonic vocalizations, which are arbitrarily defined based on human auditory perception, correlate with the number of sonic vocalizations, as previously most studies have focused only on USVs. Mice can discriminate simple versus complex USVs [53], but it is still unknown whether they can discriminate among the various other types of USVs.

### Statistical analyses

Statistical analyses were conducted using IBM SPSS Statistics 25, R 3.5.0 and R 3.6.2 and we provide means and s.e.m., unless stated otherwise. We used following working definitions and analyses:

#### Vocalization rate

The total number of vocalizations (sonic or/and ultrasonic) counted in each 10 min file. All *sonic* vocalizations were manually labelled in each 10 min file. All analysed vocalizations were confirmed by visual and/or acoustic inspection during manual classification.

#### Spectro-temporal features of USVs

Vocal spectro-temporal features were automatically calculated by A-MUD. All unclassified vocalizations were excluded from these analyses due to their noisy spectrographic features, even though they could be confirmed as USVs by auditory inspections. One mouse in the 10d group did not emit any vocalization and one mouse in the 30d group only emitted one unclassified vocalization. Thus, no vocal spectro-temporal parameters were calculated for these two mice. We analysed *length* (ms), *mean frequency* (kHz), *mean amplitude* (dB) and *slope* (kHz/ms) of each USV during each 10 min trial. The slope was automatically calculated by linear regression using all points in the detected frequency track (kHz/ms). The slope of the resulting regression line is a simplified approximation of the frequency evolution over time and needs to be interpreted with caution (see S1 File).

The statistical analyses of vocalization rates and spectro-temporal features of USVs were computed using R 3.6.2 [57]. Vocalization rate of all vocalizations and USVs showed a negative binomial distribution and were analysed using function glm.nb from package MASS [58]. There were no signs of overdispersion (dispersion parameter 0.95 and 0.72, respectively). The distribution of all model residuals was visually inspected using package fitdistrplus [59]. Residuals from models for the response variable “latency to vocalize” were approximately normally distributed, and therefore, latency was analysed using function lm.

Data on spectro-temporal features of USVs included repeated measurements, and were analysed using generalized linear mixed effects (GLMM) models (package lme4) [60]. We always used animal identity to compute random intercepts. Applied distribution families were inverse gaussian (USV length, slope and frequency), and gaussian (amplitude). For all response variables, we computed three models: First, a null model with an intercept only (describing the data by their mean). Second, a “treatment” model, comparing controls with all primed animals, irrespective of the time since priming. Third, a “timepoints” model with 5 groups, i.e., before priming and day 1, day 10, day 20 and day 30 after priming. We compared these models using AICc, Akaike’s Information Criterion corrected for small sample sizes, computed using package MuMIn [61]. To directly assess the support for each candidate model, we also computed relative model likelihoods from the differences in AICc to the best model (ΔAICc) as relative likelihood = exp(-0.5 * ΔAICc) [62]. We subsequently provide p-values for the effects from the best model, following Zuur et al (2009) [63]. We always used so-called “treatment contrasts” by placing the control group on the intercept, and then comparing all other groups to these unprimed males. We did not compute post-hoc comparisons. The letters in the figures refer to the GLM/GLMM regression coefficients, i.e., the significance comparing differences between day 0 versus any later day (in the case of “timepoints” models), and to differences between “unprimed” and “primed” (in the case of “treatment” models). In one case (variable mean frequency), the variability in the data noticeably decreased after priming. To analyse these changes in variability among USV frequencies, we computed the absolute deviation of frequency measurements from their median as the response variable, analogous the Levene’s test, and computed a GLMM, again with random intercepts per animal.

#### Vocal repertoire of USVs

Vocal repertoire was assessed using two measures. First, repertoire *diversity*, which is total number of vocalization types per sound-file (Fig 1). This number is a rough estimate of diversity and ranges from 0 (no vocalization) to 15 (maximal amount of diversity). Variables “repertoire diversity” and “repertoire diversity without unclassified vocalizations” were Poisson distributed and analysed with glm. There were no signs of overdisperison (dispersion parameters 0.872 and 0.878, respectively). Second, to investigate the number of vocalizations per each type, i.e., vocalization type occurrence, we calculated repertoire *composition*. We analysed repertoire composition using a multivariate approach by running a non-parametric analysis of similarities (ANOSIM) in R (V 3.5.0) [64] (package “vegan”, functions “vegdist” and “anosim”) [65]. The ANOSIM statistic compares the mean of ranked dissimilarities between groups to the mean of ranked dissimilarities within groups. The generated R value lies between -1 and +1, with a value of 0 representing the Null hypothesis (indistinguishable groups), an R close to 1 indicates that dissimilarity between groups are greater than within groups, while an R values < 0 indicate that dissimilarities within groups are greater than between groups. The test was run with 999 permutations and using the Bray-Curtis dissimilarity matrix. We also ran a permutational multivariate analysis of variance (PERMANOVA) with 999 permutations in R (package “vegan”, function “adonis2”) [65]. The calculated pseudo F-ratio compares the total sum of squared dissimilarities among vocalizations of different groups to vocalizations within the same group. Larger F-ratios indicate pronounced group separation. The one mouse in the 10d group, which did not emit any vocalizations was excluded from these analyses. To visualize the results, we used a non-metric multidimensional scaling (nMDS) approach based on the Bray-Curtis dissimilarity index (package “vegan”, function “metaMDS”). A stress coefficient of <0.05 indicates an excellent visualization of data, whereas a stress coefficient of >0.3 indicates an almost arbitrary position of data on the graph [66]. Our stress values were calculated as the mean of the 21 iterations we ran. The similarity between groups is measured by the distance between the points: the closer the distance, the greater the similarity in the composition of the vocal repertoire between groups. We also investigated vocalization type contribution to group dissimilarities using the “simper” function in R, which performs pairwise comparisons of groups to find the average contribution of each vocalization type to the average overall Bray-Curtis dissimilarity, displaying the most important vocalization types for each pair of groups. These vocalization types contributed at least to 70% of the differences between the groups. We also visualized the vocal repertoire in pie charts by calculating the proportions of each vocalization type for each mouse and then averaging these proportions for each experimental group.

### Ethical statement

After the recordings, all the mice were returned to their home cages and kept in our colony. This study was carried out in strict accordance with the recommendations in the Guide for the Care and Use of Laboratory Animals of the National Institutes of Health and complies with the current laws of Austria. All the experiments were conducted at the Konrad Lorenz Institute of Ethology, Austria and the protocols have been approved and were in accordance with ethical standards and guidelines in the care and use of experimental animals of the Ethical and Animal Welfare Commission of the University of Veterinary Medicine, Vienna (Austria) (ETK-17/04/2015) in accordance with Good Scientific Practice guidelines and national legislation. We did not sacrifice any of the mice used for this study.

## Results

### Vocalization rate (ultrasonic and sonic)

#### USVs

**Fig 2:**
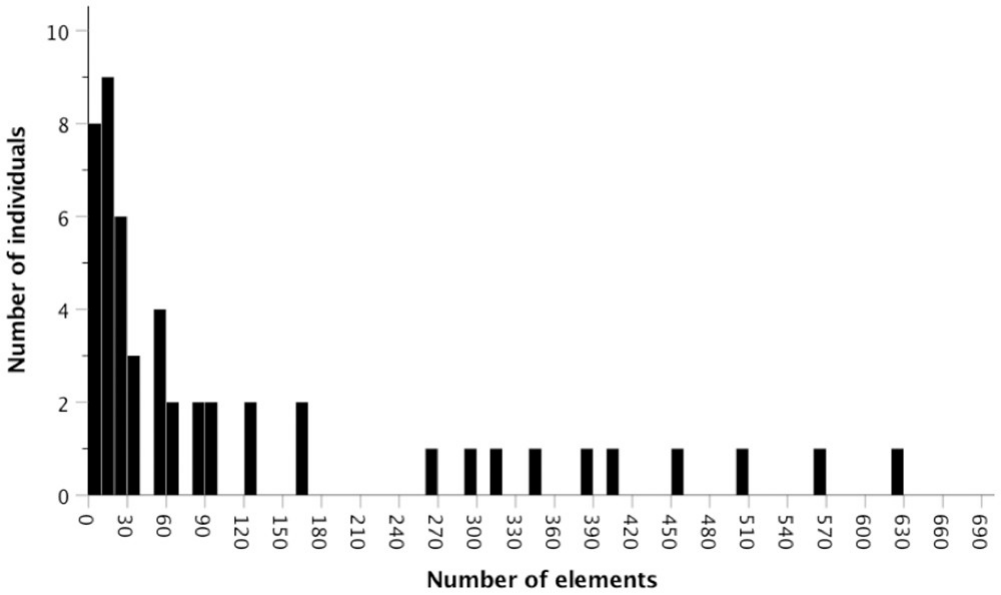
Histogram depicting variation in ultrasonic vocalization rate (number of USVs per 10 min) among individuals. Approximately half of the males emitted less than 50 USVs during the 10 min trials, though some mice were very vocal (n = 50).

**Fig 3:**
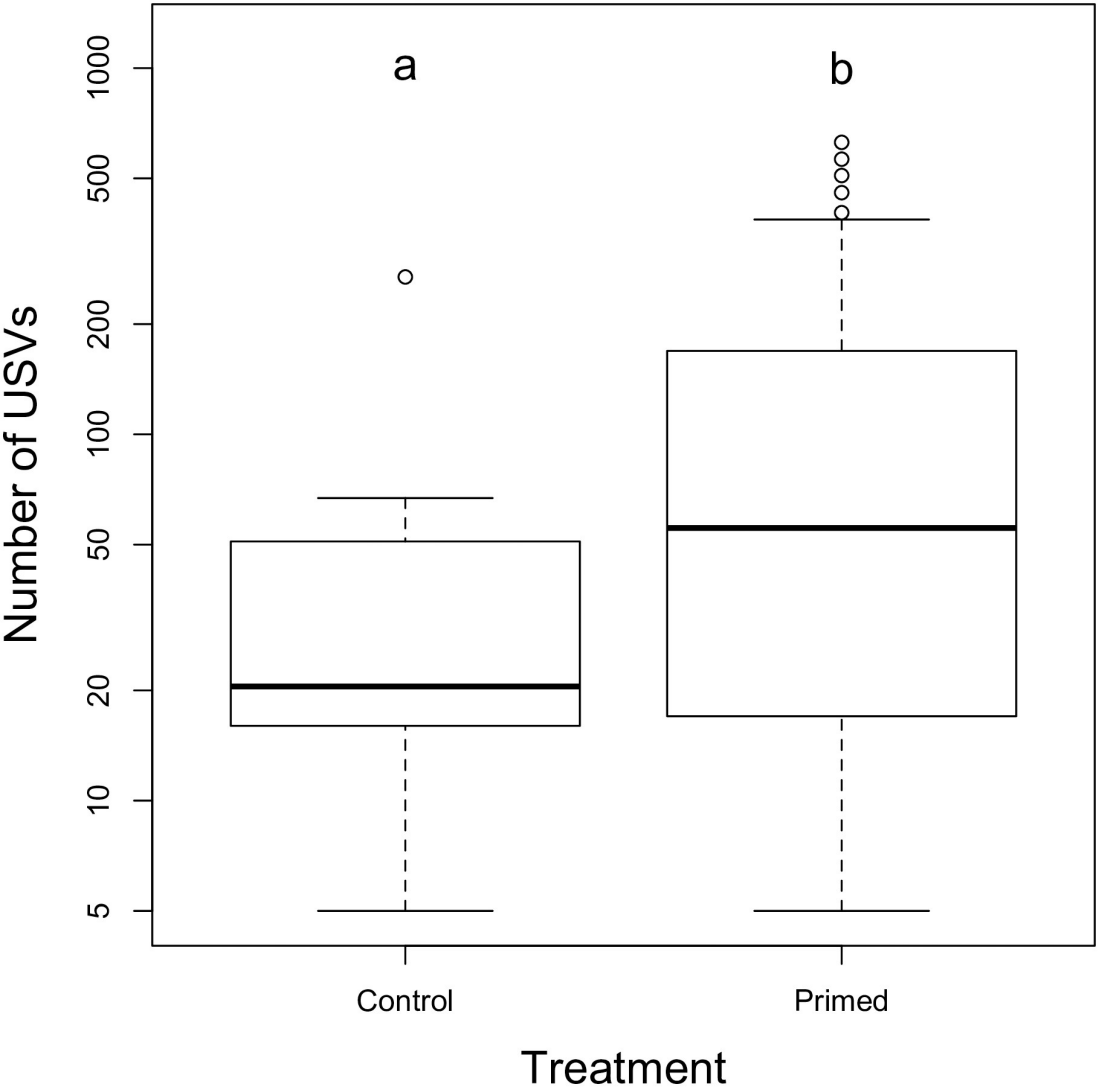
Number of USVs emitted with or without sexual priming. Boxplots of the number of USVs emitted by unprimed (control) and primed males. Boxes around the median (horizontal line) show the interquartile range (quartile 1 to 3) and whiskers extend to 1.5 times this range, or to the most extreme point, whichever is closer to the median. Extreme points are shown as circles. Different letters denote significant differences (p<0.05).

The number of USVs emitted per individual was highly variable, ranging from 0 to 627 USVs with an overall mean±s.d. of 117±164 vocalizations per male during the 10 min recordings (median = 36 vocalizations/10 min). The USV count was right skewed: approximately half of the males emitted ≤ 50 USVs (n = 26) and the other half emitted between 51–627 USVs (n = 24) (Fig 2). The mean number of USVs increased from 50±25 among unprimed control males to 142±29 among primed males (negative binomial GLM; p = 0.013, Fig 3, Table 1). The model comparing treatments had a lower AICc (554.25) than both the null model (AICc = 556.76) and the timepoints model (AICc = 560.49).

**Table 1:** Tables 1 to 5 are regression tables of effects of priming on various aspects of vocalization in male mice.

Number of USVs (GLM negative binomial)					
Coefficients:					
	Estimate	Std. Error	z value	P value	Relative Likelihood
**model timepoints** AICc = 560.49					0.04
(Intercept)	3.92	0.37	10.6	<2e-16 ***	
groupa1	1.22	0.52	2.3	0.02 *	
groupa10	0.63	0.54	1.2	0.24	
groupa20	1.10	0.52	2.1	0.03 *	
groupa30	1.07	0.54	2.0	0.05 *	
**model treatment AICc = 554.25**					1.0
(Intercept)	3.92	0.37	10.5	<2e-16 ***	
treatPrimed	1.04	0.42	2.5	0.01 *	
**Null model** AICc = 556.76					0.28

In all models, intercepts represent the mean of the control group. Coefficient estimates are the differences of group means to the intercept (at 1, 10, 20, and 30 days after priming for timepoint models, and for pooled data after priming for treatment models). Tables also show standard errors, t-values, z-values and P-values for the deviation of these differences from zero. Null models are intercept only models. AICc values give Akaike’s Information Criterion corrected for small sample size. The best AICc values are printed in bold face and relative likelihoods are the plausibilities of candidate models compared with the best model. The model type is given in parentheses. For mixed models (adjusting for repeated measurements) the random effects, e.g., standard deviations of intercepts of individuals, are also provided. Signif. codes: 0 ‘***’ 0.001 ‘**’ 0.01 ‘*’ 0.05 ‘.’ 0.1 ‘ ‘ 1.

On average, it took the mice 120±136 s before emitting the first USV (“latency to vocalize”), with a minimum = 0.03 s to a maximum = 600 s (no vocalizations, n = 1) and group means±s.d. of 69.6±94 s for 0d, 64.3±117 s for 1d, 191.9±151 for 10d, 128.6±111 s for 20d and 147.3±173 s for 30d. Latency to vocalize was unaffected by priming, and the null model had the lowest AICc (Table 2). The “treatment” model was nearly undistinguishable from the null model (relative likelihood 0.63).

**Table 2:** Regression table of effects of priming on latency to call in male mice.

Latency to call (GLM gaussian)					
Coefficients:					
	Estimate	Std. Error	t value	P value	Relative Likelihood
**model timepoints** AICc = 580.29					0.15
(Intercept)	69.6	29.5	2.4	0.02 *	
groupa1	-5.3	41.7	-0.1	0.90	
groupa10	76.9	42.8	1.8	0.08	
groupa20	59.0	41.7	1.4	0.16	
groupa30	27.4	42.8	0.6	0.53	
**model treatment** AICc = 577.42					0.63
(Intercept)	70	30	2.3	0.02 *	
treatPrimed	39	34	1.2	0.25	
**Null model AICc = 576.51**					1.0

#### Sonic vocalizations

We counted number of low-frequency vocalizations (LFVs) and low-frequency harmonic vocalizations (squeaks) to investigate whether USVs correlate with sonic vocalizations and are a good representation of a male overall vocalizations (i.e., sonic and ultrasonic) as explained above. We found that LFVs showed high individual variability (overall mean±s.d.: 9.9±8 and range: 0–46; means per group: 0d = 7.3±5, 1d = 16±13, 10d = 7.6±6, 20d = 9.5±6 and 30d = 9.2±8). The number of LFVs and ultrasonic vocalizations positively correlated with each other (Spearman correlation: ρ = 0.49, n = 50, p<0.0001). Squeaks were also highly variable (overall mean±s.d.: 11.9±21.2 and range: 0–132; means per group: 0d = 17.6±40, 1d = 17.7±18, 10d = 2.9±3, 20d = 12.4±10 and 30d = 8.9±15) and positively correlated with USVs as well (Spearman correlation: ρ = 0.469, n = 50, p = 0.001). Taken together, USVs positively correlated with sonic vocalizations (Spearman correlation: ρ = 0.502, n = 50, p = 0.0001) and thus we also investigated priming effects on overall vocalization rates, as there were too few sonic vocalizations to be analysed separately and draw meaningful conclusions.

#### Sonic and ultrasonic vocalizations

**Fig 4:**
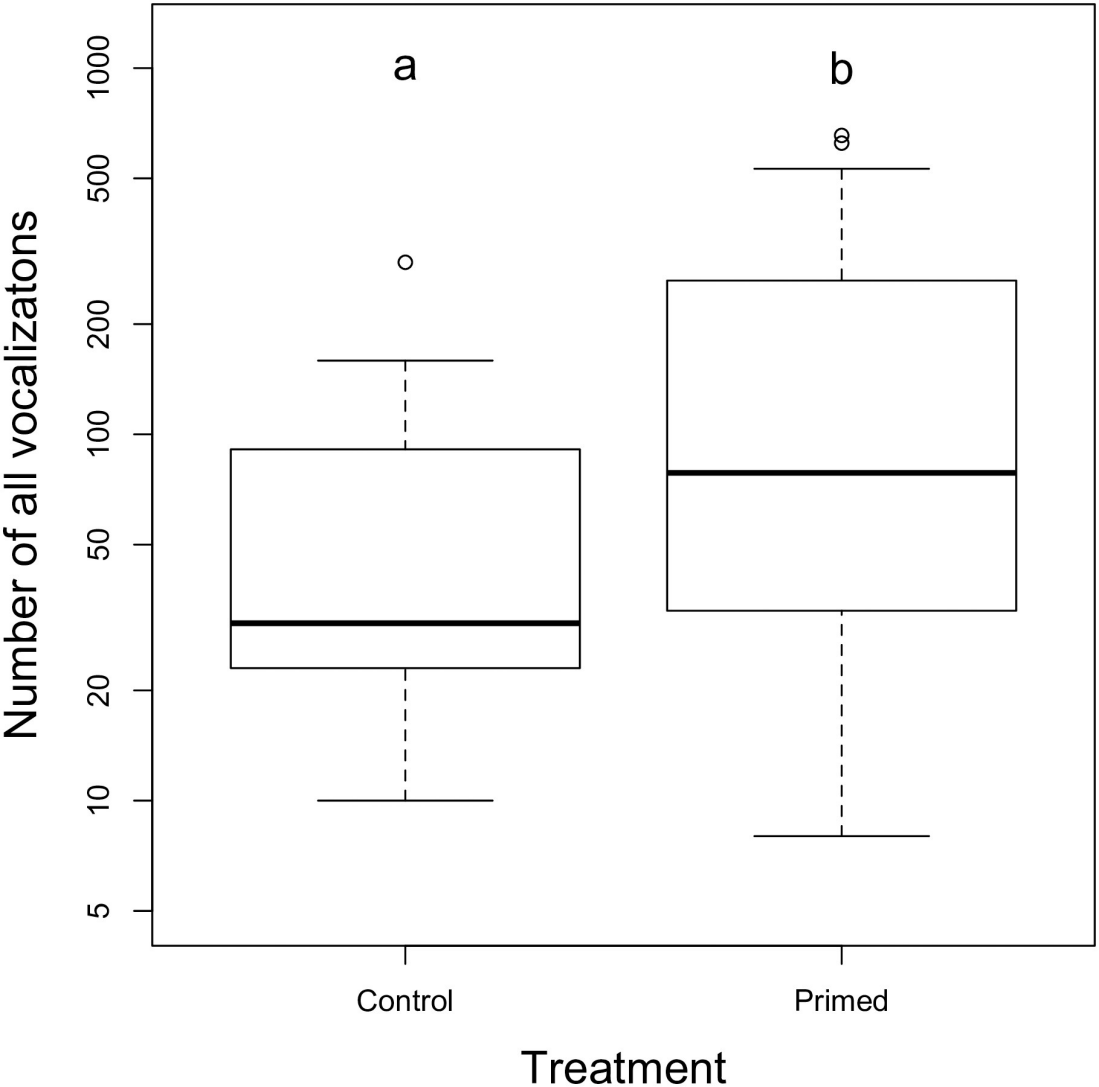
Number of all vocalizations (sonic and ultrasonic) emitted with or without sexual priming. Boxplots with medians of the number of the overall vocalizations emitted by unprimed (control) and primed males. Different letters denote significant differences (p<0.05).

We investigated priming effects on the overall vocalization rates, i.e., merging ultrasonic and sonic vocalizations. The mean number of all vocalizations increased from 75.1±28.4 to 163.7±29.8 (controls vs primed males; negative binomial GLM; p = 0.036, Fig 4). This treatment model (AICc = 576.57) was slightly better than the null model (intercept only; AICc = 577.95, relative likelihood 0.5), but much better than a model differentiating between timepoints (AICc = 582.14, Table 3).

**Table 3:** Regression table of effects of priming on number of all vocalizations in male mice.

Number of all vocalizations (GLM negative binomial)					
Coefficients:					
	Estimate	Std. Error	z value	P value	Relative Likelihood
**model timepoints** AICc = 582.14					0.06
(Intercept)	4.32	0.33	13.3	<2e-16 ***	
groupa1	1.00	0.46	2.2	0.03 *	
groupa10	0.34	0.47	0.7	0.47	
groupa20	0.84	0.46	1.8	0.07	
groupa30	0.80	0.47	1.7	0.09	
**model treatment AICc = 576.57**					1.0
(Intercept)	4.32	0.33	13.0	<2e-16 ***	
treatPrimed	0.78	0.37	2.1	0.04 *	
**Null model** AICc = 577.95					0.50

### Spectro-temporal features of USVs

We calculated the spectro-temporal features of all detected USVs omitting uc vocalizations due to their noisy and unstructured features, n = 5151 USVs. Frequency, slope and amplitude parameters could not be calculated for 198 USVs due to being too short or faint.

**Fig 5:**
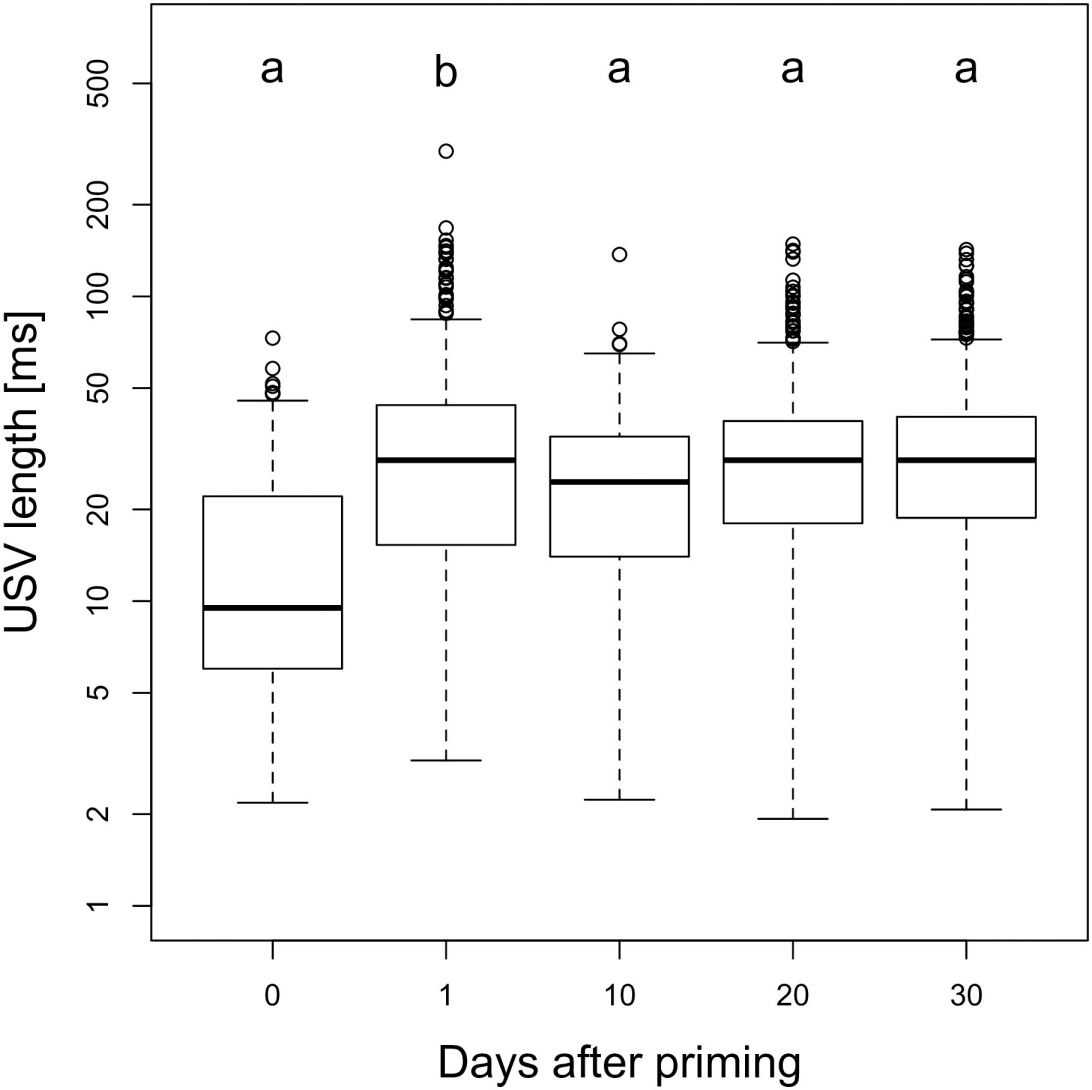
USV length with or without priming. Boxplots with medians of USV lengths emitted by unprimed (0) and primed males (≥1). Different letters denote significant differences (p<0.05).

**Fig 6:**
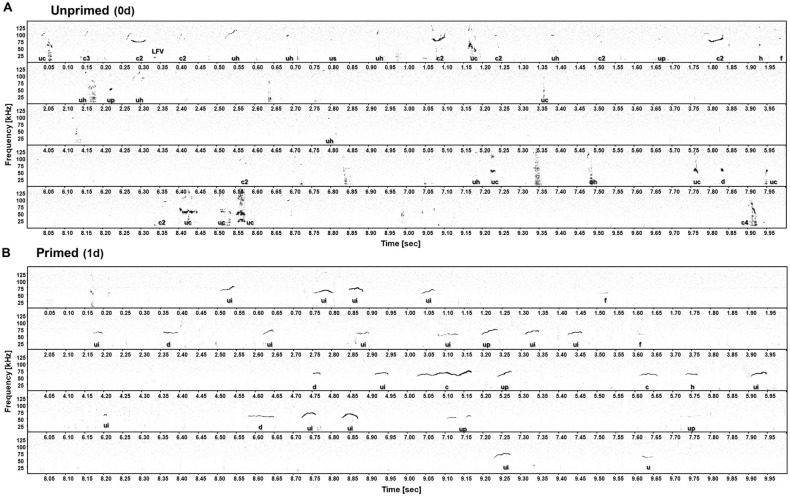
Spectrogram examples of an unprimed (0d) and a sexually primed (1d) male. The two spectrograms show a 10 s continuous sequence of the males that emitted most USVs in both groups, (A) the unprimed group and (B) in the group recorded 1d after priming. All lines of the spectrograms are continuous and each line shows 2 s (50 ms interval) of the 10 s sequence. Y-axes represent frequencies between 0–150 kHz with intervals of 25 kHz. Letters indicate examples of vocalization types, following the definitions and abbreviations in Fig 1. LFV = low-frequency vocalization.

**Fig 7:**
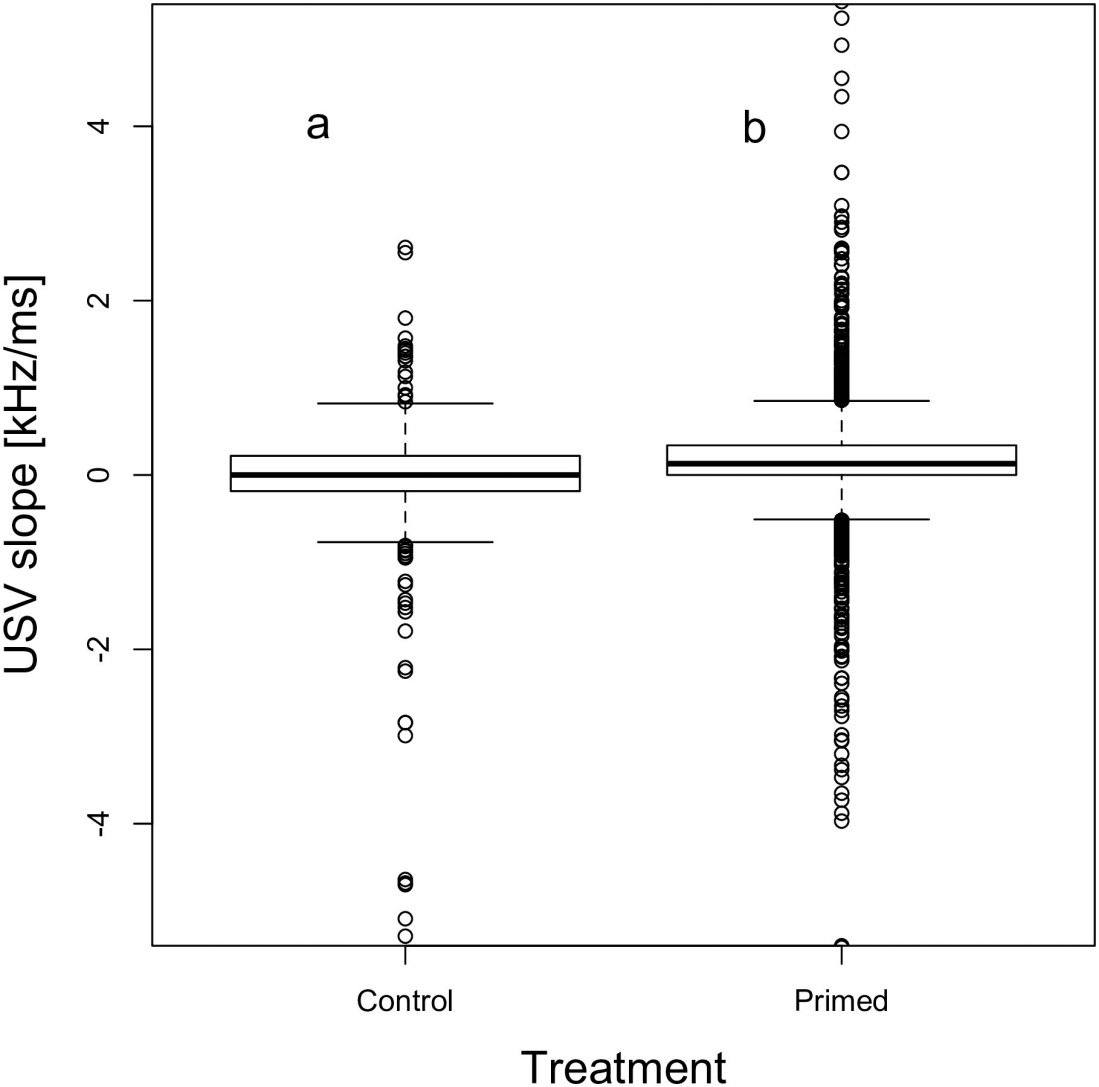
USV slope with or without priming. Boxplots with medians of USV slopes emitted by unprimed (control) and primed males. Different letters denote significant differences (p<0.05).

**Fig 8:**
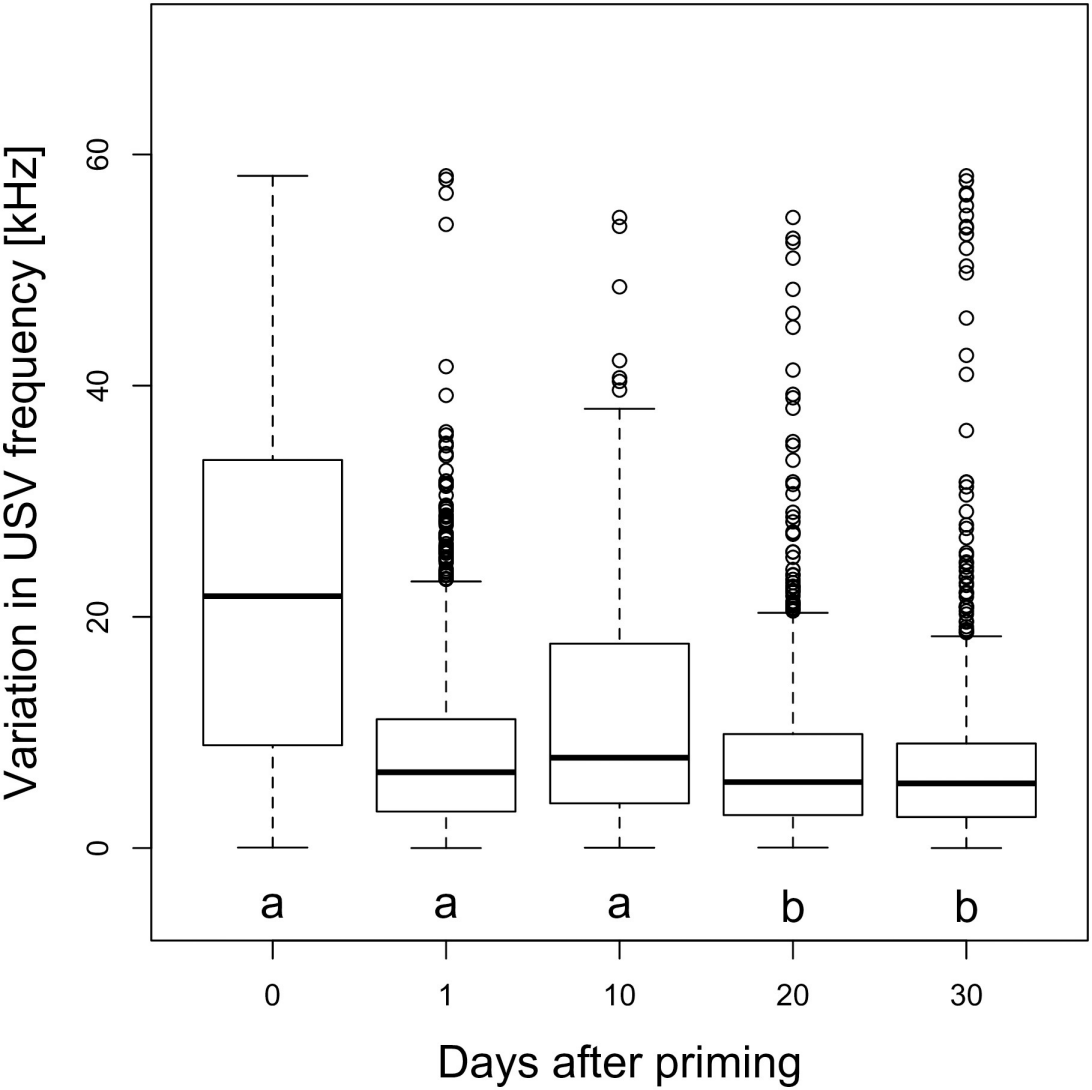
Variability in USV frequency between primed and unprimed males. Boxplots of absolute deviations of the USV frequency from the overall median. Different letters denote significant differences (p<0.05).

The mean length of USVs more than doubled at day 1 after priming (14.93±0.71 ms vs. 32.5±0.60 ms; p = 0.006, Figs 5 and 6). GLMM analysis indicated that USV length was significantly longer after priming at day 1 (p = 0.006), but not on later trial dates. The model differentiating between timepoints (AICc = 43344.88) was better than both the null model (AICc = 43347.68) and the treatment model (AICc = 43348.74, Table 4). USVs’ slope increased from -0.16±0.11 in unprimed to +0.16±0.01 in primed animals (GLMM; p = 0.022, Fig 7). This model comparing treatments had the lowest AICc (-27972.49). The model differentiating between all timepoints (AICc = -27969.47) and the null model (AICc = -27969.86) were inferior (Table 4). The mean frequency (kHz) of male vocalizations was unaffected by priming (GLMM; p>0.3); however, there was lower variability in the vocalizations of primed compared to unprimed males (Table 4). This decrease in the variability of frequency of primed males was a tendency at day 1 (p = 0.051), and significant on days 20 (p = 0.029) and 30 (p = 0.026, Fig 8). Neither the magnitude nor the variability of USV amplitude were affected by priming (Table 4).

**Table 4:** Regression table of effects of priming on the spectro-temporal features of USVs in male mice.

**Spectro-temporal features of USVs: Length (GLMM)**							Relative Likelihood
**model timepoints AICc = 43344.88**							1.0
Random effects:							
Groups	Name		Variance		Std.Dev.		
Replicate	(Intercept)		3.4e-06		0.0019		
Residual			1.6e-02		0.1269		
Number of obs: 5151, groups: Replicate, 48							
Fixed effects:							
	Estimate	Std. Error		t value	P value		
(Intercept)	0.0121	0.0019		6.5	9e-11 ***		
groupa1	-0.0080	0.0029		-2.7	0.006 **		
groupa10	0.0016	0.0025		0.6	0.541		
groupa20	-0.0025	0.0026		-1.0	0.336		
groupa30	-0.0015	0.0030		-0.5	0.607		
**model treatment** AICc = 43348.74							0.15
Random effects:							
Groups	Name		Variance		Std.Dev.		
Replicate	(Intercept)		4.8e-06		0.0022		
Residual			1.6e-02		0.1272		
Number of obs: 5151, groups: Replicate, 48							
Fixed effects:							
	Estimate	Std. Error		t value	P value		
(Intercept)	0.0129	0.0021		6.1	1e-09 ***		
treatPrimed	-0.0023	0.0023		-1.0	0.3		
**Null model** AICc = 43347.68							0.25
**Spectro-temporal features of USVs: slope (GLMM)**							Relative Likelihood
**model timepoints** AICc = -27969.47							0.21
Random effects:							
Groups	Name		Variance		Std.Dev.		
Replicate	(Intercept)		0.0020		0.045		
Residual			0.0039		0.062		
Number of obs: 4948, groups: Replicate, 48							
Fixed effects:							
	Estimate	Std. Error			t value	P value	
(Intercept)	16.20	0.14			119.0	<2e-16 ***	
groupa1	-0.29	0.16			-1.9	0.064	
groupa10	-0.30	0.17			-1.7	0.081	
groupa20	-0.42	0.16			-2.6	0.008 **	
groupa30	-0.47	0.16			-2.9	0.004 **	
**model treatment AICc = -27972.49**							1.0
Random effects:							
Groups	Name		Variance		Std.Dev.		
Replicate	(Intercept)		0.0030		0.055		
Residual			0.0039		0.062		
Number of obs: 4948, groups: Replicate, 48							
Fixed effects:							
	Estimate	Std. Error			t value	P value	
(Intercept)	16.20	0.15			110.1	<2e-16 ***	
treatPrimed	-0.36	0.16			-2.3	0.02 *	
**Null model** AICc = -27969.86							0.23
**Spectro-temporal features of USVs: frequency (GLMM)**							Relative Likelihood
**model timepoints** AICc = -8862.94							<0.01
Random effects:							
Groups	Name		Variance		Std.Dev.		
Replicate	(Intercept)		0.308		0.55		
Residual			0.044		0.21		
Number of obs: 4948, groups: Replicate, 48							
Fixed effects:							
	Estimate	Std. Error			t value	P value	
(Intercept)	4.06	0.40			10.2	<2e-16 ***	
groupa1	-0.85	0.68			-1.3	0.2	
groupa10	-0.49	0.59			-0.8	0.4	
groupa20	-0.44	0.60			-0.7	0.5	
groupa30	-0.38	0.64			-0.6	0.6	
**model treatment** AICc = -8868.47							<0.01
Random effects:							
Groups	Name		Variance		Std.Dev.		
Replicate	(Intercept)		0.308		0.56		
Residual			0.044		0.21		
Number of obs: 4948, groups: Replicate, 48							
Fixed effects:							
	Estimate	Std. Error			t value	P value	
(Intercept)	4.06	0.40			10.2	<2e-16 ***	
treatPrimed	-0.52	0.46			-1.1	0.3	
**Null model AICc = -8889.24**							1.0
**Spectro-temporal features of USVs: variability of frequencies (GLMM)**							Relative Likelihood
**model timepoints** AICc = 30480.26							0.23
Random effects:							
Groups	Name		Variance		Std.Dev.		
Replicate	(Intercept)		0.00083		0.029		
Residual			0.60243		0.776		
Number of obs: 4948, groups: Replicate, 48							
Fixed effects:							
	Estimate	Std. Error			t value	P value	
(Intercept)	0.059	0.012			4.8	2e-06 ***	
groupa1	0.033	0.017			1.9	0.05	
groupa10	0.011	0.017			0.6	0.52	
groupa20	0.037	0.017			2.2	0.03 *	
groupa30	0.039	0.017			2.2	0.03 *	
**model treatment AICc = 30477.29**							1.0
Random effects:							
Groups	Name		Variance		Std.Dev.		
Replicate	(Intercept)		0.00089		0.03		
Residual			0.60263		0.78		
Number of obs: 4948, groups: Replicate, 48							
Fixed effects:							
	Estimate	Std. Error			t value	P value	
(Intercept)	0.060	0.013			4.7	3e-06 ***	
treatPrimed	0.030	0.014			2.1	0.03 *	
**Null model** AICc = 30479.30							0.36
**Spectro-temporal features of USVs: amplitude (GLMM)**							Relative Likelihood
**model timepoints AICc = 26662.96**							1.0
Random effects:							
Groups	Name		Variance		Std.Dev.		
Replicate	(Intercept)		2.3		1.5		
Residual			12.6		3.5		
Number of obs: 4948, groups: Replicate, 48							
Fixed effects:							
	Estimate		Std. Error		t value		
(Intercept)	15.316		0.664		23.0		
groupa1	0.878		0.838		1.0		
groupa10	-0.242		0.898		-0.3		
groupa20	0.048		0.876		0.1		
groupa30	-0.478		0.873		-0.5		
**model treatment** AICc = 26664.09							0.56
Random effects:							
Groups	Name		Variance		Std.Dev.		
Replicate	(Intercept)		2.3		1.5		
Residual			12.6		3.5		
Number of obs: 4948, groups: Replicate, 48							
Fixed effects:							
	Estimate		Std. Error		t value		
(Intercept)	15.32		0.67		22.9		
treatPrimed	0.10		0.73		0.1		
**Null model** AICc = 26663.3							0.84

### Vocal repertoire of USVs (repertoire diversity and composition)

We found that number of USVs were positively correlated with repertoire diversity (Spearman correlation: ρ = 0.91, n = 50, p<0.0001). GLM analysis showed that the timepoint model for repertoire diversity had the lowest AICc (AICc = 289.68), but the treatment model was nearly undistinguishable (AICc = 290.26, relative likelihood 0.74); the null model was clearly worse (AICc = 294.01, Table 5).

**Table 5:** Regression table of effects of priming on the vocal repertoire in male mice.

Vocal repertoire (GLM Poisson)					Relative Likelihood
Coefficients:					
	Estimate	Std. Error	z value	P value	
**model timepoints AICc = 289.68**					1.0
(Intercept)	1.758	0.131	13.4	<2e-16 ***	
groupa1	0.525	0.166	3.2	0.002 **	
groupa10	0.083	0.182	0.5	0.650	
groupa20	0.346	0.172	2.0	0.044 *	
groupa30	0.334	0.172	1.9	0.052	
**model treatment** AICc = 290.26					0.74
(Intercept)	1.76	0.13	13.4	<2e-16 ***	
treatPrimed	0.33	0.14	2.3	0.02 *	
**Null model** AICc = 294.01					0.11

**Fig 9:**
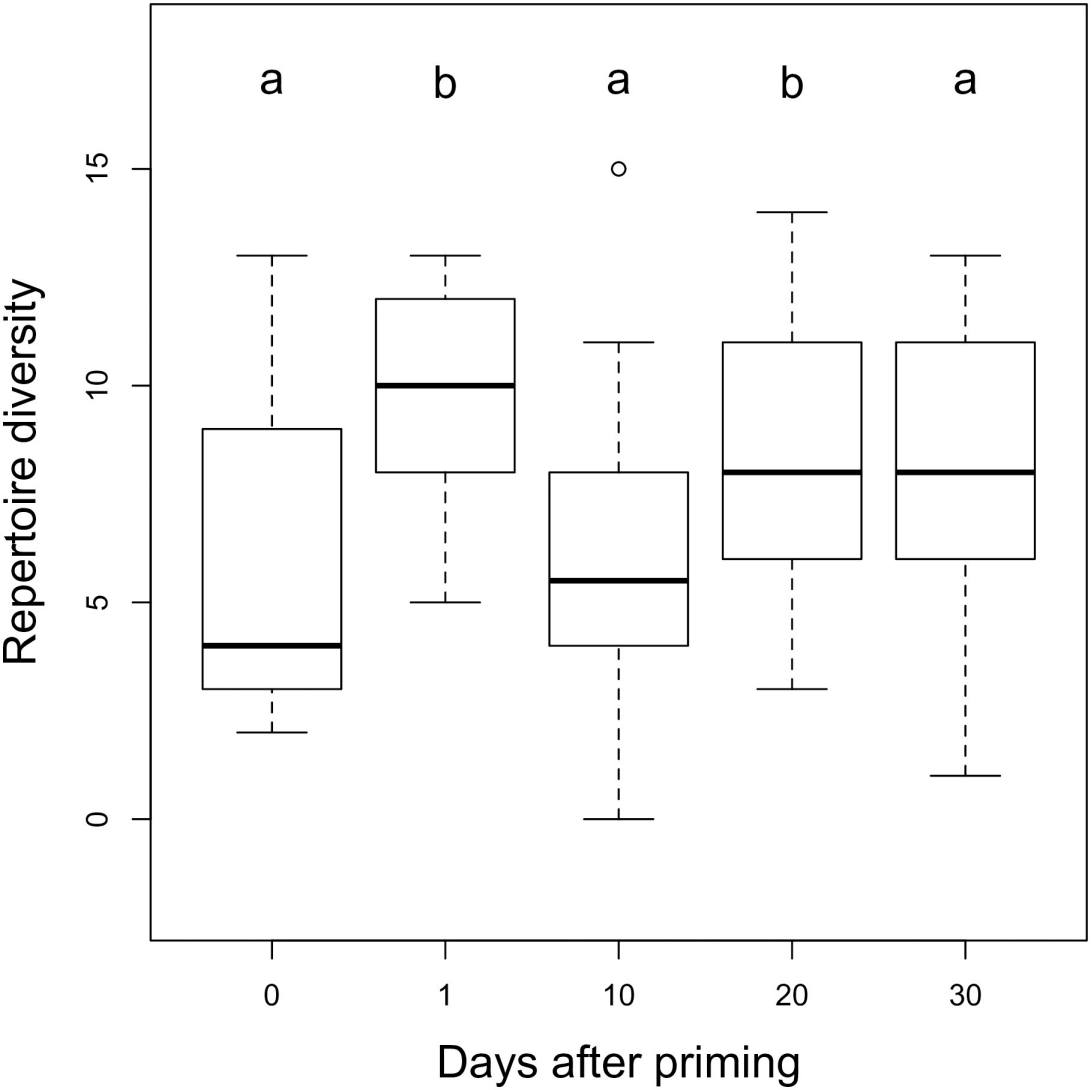
Repertoire diversity of USV with or without priming. Boxplots with medians of repertoire diversity of unprimed (0) and primed males (≥1). Different letters denote significant differences (p<0.05).

Compared with controls, repertoire diversity was significantly increased at days 1 (p<0.002) and 20 (p<0.044) after priming (Fig 9).

**Fig 10:**
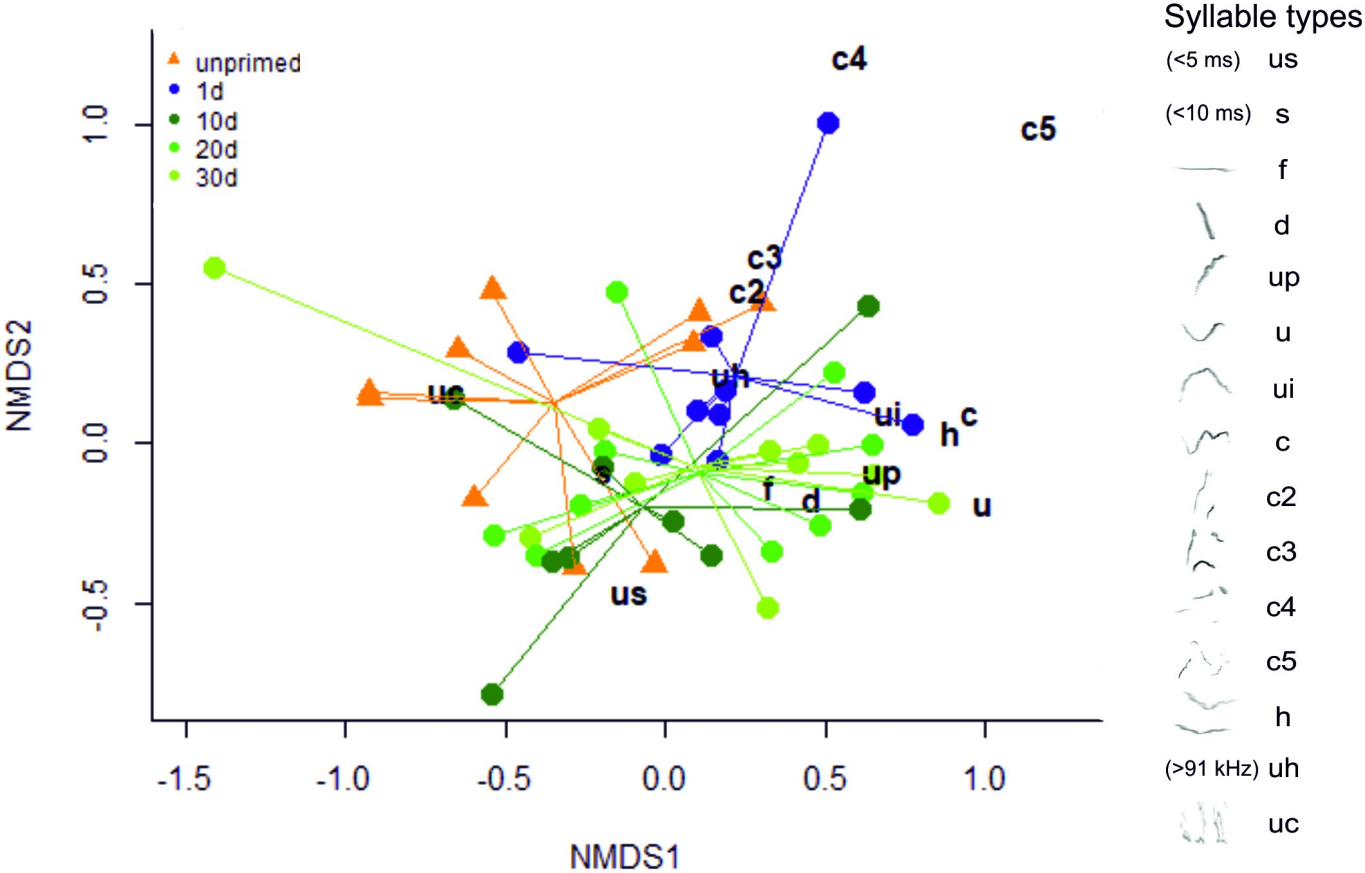
Non-metric multi-dimensional clustering of USV type according to priming groups (nMDS: Stress = 0.13). Mice are clustered according to the amount of each vocalization type emitted, and spectrograms of vocalization types are depicted on the legend on the right (see also Fig 1). Mice are color-coded by groups: unprimed males in orange triangles, 1d primed males in blue circles, 10d primed males in dark green circles, 20d primed males in green circles and 30d primed males in light green circles.

**Fig 11:**
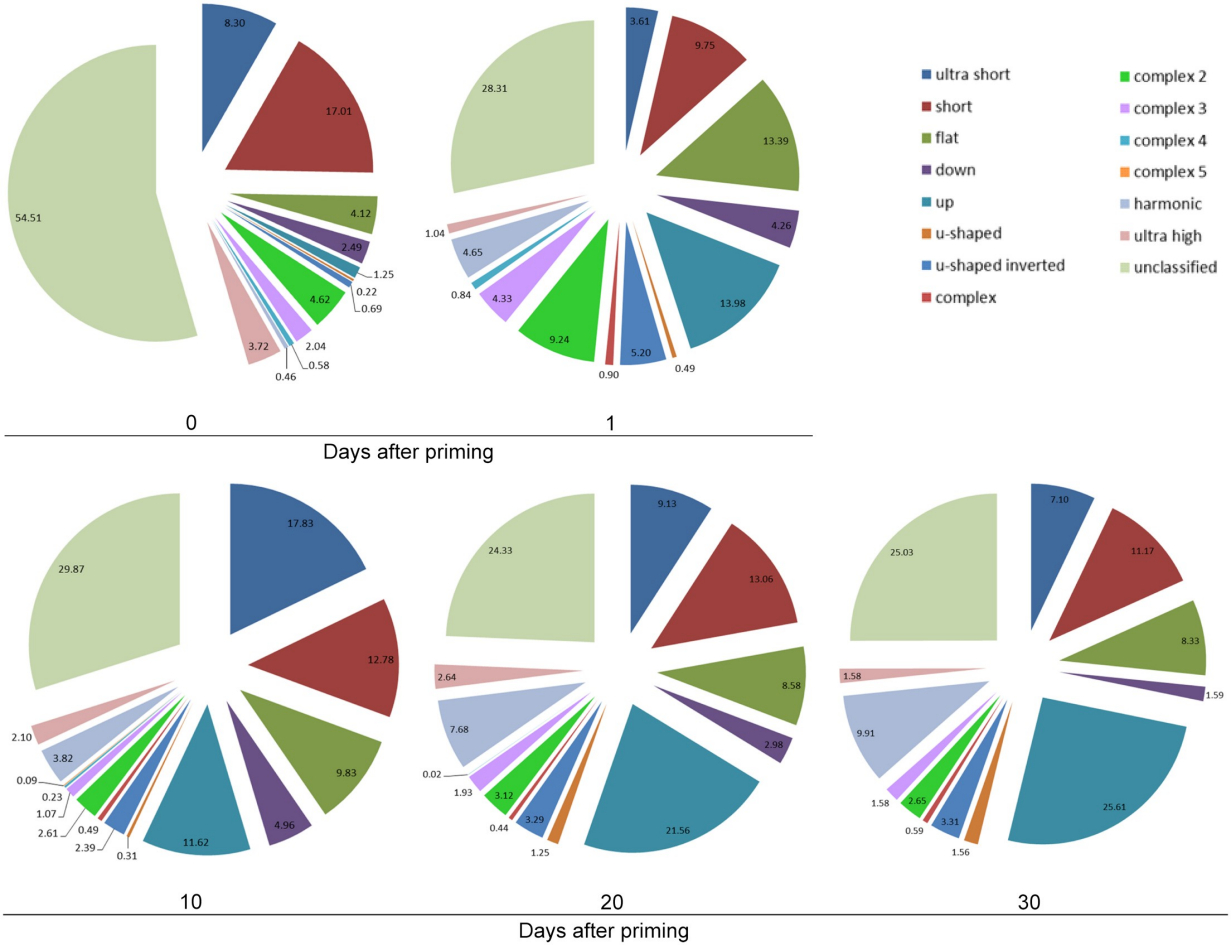
Proportions of the different types of vocalizations emitted by mice in the treatment and control groups. Pie charts show the mean proportions (%) of the occurrence of vocalization types emitted by each group, and the legend shows the 15 vocalization types (see also Fig 1).

We examined priming effects in repertoire composition to assess vocalization type occurrence per group, using two statistical non-parametric multivariate approaches. Both analyses showed that groups differed and that the unprimed males had the most distinctive vocal repertoire (ANOSIM: R = 0.108, p = 0.01) and (PERMANOVA: F(4,44) = 1.98, p = 0.015). We visualized the data using non-metric multi-dimensional clustering (nMDS), and plotted the occurrence of each vocalization type per experimental group (Fig 10). This graph shows the clustering of the vocalization types emitted by individuals (coloured symbols) to visualize the differences within versus between groups (individuals are connected to a centroid that minimizes the distances between individuals within each group). The different vocalization types (represented by letters) are positioned according to their highest clustering. The main difference appears to be due to unprimed males emitting more unclassified (uc) and fewer complex vocalizations than the other mice, whereas the 1d primed males have more ultra-high (uh), complex and less short (s) and ultra-short (us) vocalizations than the other mice (Fig 10). Further visualization of the proportions of vocalization types emitted by mice in the different groups are shown in pie charts (Fig 11), which indicate that the main differences were between the primed and unprimed males.

We also conducted the same analyses omitting all unclassified USVs, because, although confirmed by auditory inspection as vocalizations, they appeared unstructured and noisy compared to the other USVs. We found that the results on repertoire diversity are largely unchanged when omitting unclassified (uc) USVs. The nMDS graph displaying repertoire composition shows a separation of the 0d and 1d group, which is now mainly driven by short USVs (instead of the omitted uc) (see S1 Results in S1 File and S5-S7 Figs in S1 File).

## Discussion

Our main aim was to experimentally test whether male house mice show increased rates of vocalizing following a direct interaction with an adult female (sexual priming), and our most important results include the following: First, we found that sexually primed males emitted significantly more USVs than unprimed controls, consistent with previous studies measuring 70 kHz vocalizations of laboratory mice [e.g. 39]. We also found that the rates of ultrasonic and sonic vocalizations were positively correlated with each other. Thus, USVs in our study provided a good estimate of the rates of sonic calls and the overall vocalization rates, and these relationships have not been previously compared to our knowledge. The effect of priming on overall vocalization rates were likely dominated by USVs, however, because sonic vocalizations were less common.

Second, we developed an improved version of automated USV detection (A-MUD, version 3.2) and we used the data from this study to evaluate its performance. We found that lowering the detection threshold reduced false negative error rates, but it also increased the risk of false positives. This detection trade-off was acceptable because we also manually classified the vocalizations in this study, and thus we were able to correct the output as necessary. This new version of A-MUD (3.2) includes a *quality evaluation score* for each detected element (an estimate in the confidence of a true positive) and it also enables users to remove segments below a certain criterion. We evaluated A-MUD’s performance using receiver operating characteristic (ROC) curve and found that the AUC value was excellent. This tool is free for scientific (non-profit) use and available here: https://www.kfs.oeaw.ac.at/doc/amud/AMUD1b.sts (Script); Readme: https://www.kfs.oeaw.ac.at/doc/amud/AMUD1b-Readme.odt.

Third, we quantified different types of vocalizations (‘syllables’), and found that the repertoire diversity of male vocalizations significantly increased one day after priming, and multivariate analyses indicated that the unprimed males had the most distinctive repertoire composition. Unprimed males emitted mostly unclassified calls and fewer complex types of vocalizations, whereas males tested one day after priming emitted more ultra-high and complex vocalizations and fewer short and ultra-short vocalizations. Vocal spectro-temporal features of USVs also differed after priming, and priming affected USV length, slope and frequency variability.

Fourth, primed males did not differ in the mean frequency (kHz) of their vocalizations from unprimed controls, but interestingly their calling frequencies showed significantly lower variability (Fig 8). As primed males showed more ‘agreement’ in their calling frequency than unprimed males, they might be ‘targeting’ female auditory perception or preferences. Future studies are needed to compare how male vocalization frequency matches female auditory sensitivity thresholds, and whether the frequency of male cUSVs influence female preferences for recorded playbacks.

Finally, the main differences in USVs were between the primed males versus the unprimed controls (treatment models), regardless of the time since priming. Since we observed changes in males’ USVs after day 1, our results provide novel evidence for long-lasting effects from sexual priming. Timepoint models indicated that USV length increased 1 day after priming, that males’ USV repertoire diversity increased 1 and 20 days after priming, and that the variability in the frequencies of vocalizations was lower 20 and 30 days after priming.

Our results could potentially be due to individual housing *reducing* the rates of USV emission of controls, and sexual priming restoring normal USV responses (though this alternative interpretation is not mutually exclusive to the hypothesis that sexual experience *increases* USV emission). Individual housing has been reported to influence the behaviour and physiology of laboratory mice in some, but not all studies [e.g. 67–69]. A recent study on laboratory mice reported that individually housed males *increased* the emission of USVs during male-male interactions compared to socially housed males [70]. Male mice were kept in either individual or social housing (4 mice per group) for five weeks, and USVs were recorded during direct interactions with other males (kept previously in individual or social housing). Interestingly, male USV emission was correlated with the male mounting behaviour of individually housed males, and the authors concluded that their findings were due to ‘inappropriate’ courtship and mating behaviour by individually housed male males towards same-sex conspecifics. If male mounting behaviour in this previous study was a consequence of sexual arousal, then our results are consistent. We have never observed male-male mounting in wild-caught or wild-derived mice, even under similar circumstances or in semi-natural conditions (wild male mice are more aggressive than most laboratory strains). Nevertheless, the effects of previous inter- and intra-sexual social experience on USV emission needs to be examined under more natural social conditions [32]. Also, we only examined the effects of previous experience of female interactions on male USVs, and future studies are needed to investigate same- versus opposite-sex priming on both sexes to determine whether such effects are sexual, social, or both (socio-sexual).

It is unclear how sexual experience induces changes in male vocalizations, but some potential neuro-endocrine mechanisms have been identified [see 39, 45, 71–73]. Sexual stimuli trigger a surge of androgens, which regulate male USV emission and other sexual behaviours [74–79]. Sexual experience induces long-term changes and selective elevations of androgen receptors in the medial preoptic area (mPOA) [80, 81], a key site for the integration of sensory inputs and control of motor behaviour, including courtship USVs [82]. Sexual priming may also influence the specialized neurons in the midbrain periaqueductal gray (PAG) that control USV emission [83]. Future studies are needed to better understand the neuro-endocrine mechanisms that control USV emission and how they are affected by sexual priming.

The functions of such experiential effects on male USV emission are also unclear. Effects from sexual priming are thought to motivate and prepare males for courtship and mating, as they also trigger increased scent-marking [84], sperm density [85], and copulatory behaviour [86]. Thus, our findings are consistent with the hypothesis that courtship USVs provide a reliable indicator of a male’s sexual arousal [20, 34, 87] [reviewed in 45, 73]. They are also consistent with a study showing that sexual priming "emboldens" male mice and increases their boldness or risk-taking [88]. Male mice have been shown to alter the amount and types of USVs they emit after they detect a female or her scent and over the course of courtship and mating [15, 43]. Thus, the changes in male USVs induced by sexual stimuli may help to attract females and enhance their receptivity. One study found that females are attracted to playbacks of male vocalizations with more complex syllable types [33], and studies are now needed to investigate how females respond to other priming-induced changes in males USVs. The dynamic changes in male courtship USV emission after a sexual encounter might provide more reliable information about a male’s identity (compatibility) or condition (quality).

In summary, our study is the first to experimentally test whether direct socio-sexual priming affects the USV emission of wild-derived house mice (*Mus musculus musculus)*, and the first to demonstrate that priming affects the repertoire diversity and composition, as well as the rate of vocalizations. We found that calls of primed males also showed altered USV spectro-temporal features, i.e., USV length, slope and variability in USV frequency. We found high individual variation in several vocalization parameters, as with previous studies of wild-derived mice (unlike many studies, we did not apply a screening procedure, such as omitting recordings of males that did not vocalize, or use threshold criteria for our analyses, as not to bias results). Given such variation, longitudinal measures are needed to further investigate priming effects on USV emission. Until then, our results suggest that USV studies should control differences in sexual priming (types of priming experience and duration after priming) as potential sources of variation.

## Supporting information

S1 File (PDF)

S1 Data (XLSX)

S2 Data (XLSX)
