# Supplementary material for: Primed to vocalize: Wild-derived male house mice increase vocalization rate and diversity after a previous encounter with a female
Source: PLoS One. 2020 Dec 9;15(12):e0242959. doi: 10.1371/journal.pone.0242959 (PMC7725367; doi:10.1371/journal.pone.0242959)
Supplement: S1 File — (PDF) [file pone.0242959.s001.pdf]

Supplementary Information

Primed to vocalize: wild-derived male house mice increase vocalization rate and diversity after a previous encounter with a female

Sarah M. Zala[1*], Doris Nicolakis[1], Maria Adelaide Marconi[1], Anton Noll[2], Thomas Ruf[3], Peter Balazs[2] and Dustin J. Penn[1]

[1] Konrad Lorenz Institute of Ethology, University of Veterinary Medicine, Vienna, 1160 Vienna, Austria.

[2] Acoustic Research Institute, Austrian Academy of Sciences, Vienna, Austria.

[3] Research Institute of Wildlife Ecology, University of Veterinary Medicine, Vienna, 1160 Vienna, Austria.

* Corresponding author:
Sarah M. Zala: sarah.zala@vetmeduni.ac.at
Konrad Lorenz Institute of Ethology, University of Veterinary Medicine, Vienna, Austria, Savoyenstraße 1, A-1160 Vienna, Austria;
Phone: +43 1 25077 7352; Fax: +43 1 25077 94 7352

S1 Methods

Development and implementation of A-MUD 1.0

The segmentation algorithm of A-MUD 1.0 was first developed and evaluated in [1]. In short:

1) We used the short-time Fourier transform with a Hanning window of length between 2.5 and 3.3 ms and an overlap between 75% and 85%.

$$STFT_w(f)[k,l] = \sum_{j=0}^{N_{FFT}-1} f[j] \cdot w[j-kH] \cdot e^{-2\pi i (j-l)l\, N_{FFT}}$$

   a. A bandpass between 30 – 120 kHz was applied, which results in restricted STFT spectra $F_k(l)$.
   b. The spectra were pre-whitened.

2) We computed the segmentation parameters by a spectral narrowness criterion *ec*, i.e. the inverse number of frequency bins for which a certain ratio of the total spectrum energy was achieved.
3) Depending on the signal, two threshold parameters were defined as 10% (‘on’) and 90% (‘off’) quantile of the above defined parameter. Local maxima of *ec* above the ‘on’ criterion are detected, and searching forward and backward (in time) until the *ec* criterion falls below ‘off’ results in segment detection.
4) Two correctional steps were added: segments with a distance below 5-10 ms are merged, and segments with a length below 5-10 ms or above 150-200 ms are discarded.

Improvements of A-MUD 3.2

The improved version used the additional steps:

1b’) To reduce the noise contribution, an exponential mean is applied for the STFT spectra $F_k(l)$, i.e. using

$$y_n = k \cdot y_{\{n-1\}} + (1-k) \cdot x_{\{n-1\}}\ for\ k \in [0,1].$$

1c) The envelope of the spectra is estimated by using 6-8 cepstral DCT coefficients [2]. This is subtracted from $F_k(l)$ and only spectral bins above it are considered in the following. The motivation for this step is that fast local changes are much more prominent, and therefore more easily detected and the absolute amplitudes become irrelevant.

The segmentation criterion is now based on a completely new parameter:

2’) Instead of the spectral narrowness in (2) the amplitudes (m1-m3) and frequencies (f1-f3) of the three highest peaks in the spectrum are measured for each time position.

3’) Two ‘on’ criteria can be used, $m1 \geq \Theta_{on}$ and $\Delta := m1 - (m2 + m3) \geq \phi_{on}$. The search for a segment starts from these positions forward and backward until the values fall below an ‘off’ criterion, i.e. $m1 \leq \Theta_{off}$ and $\Delta \leq \phi_{off}$. Note that either the first, this is the default option, or both criteria can be used. The second option should be chosen for a setting with a lot of background noise and disturbing signals.

Unlike the first version, in A-MUD 3.2 segments of 10 ms are retained, segments with length < 5ms are discarded, to remove disturbing signals like pulses, and segments >300 ms are discarded to exclude non-USVs. Note that many parameters for AMUD 3.2 can be chosen in a

dialog, and we report here the default values determined by pilot tests and used for the evaluation ($\Theta_{on} = 12\ dB, \Theta_{off} = 9\ dB,$ and $\phi_{on} = 3\ dB$ $\phi_{off} = 0\ dB$,$K = \sqrt{2}$ and 8 cepstral coefficients).

In addition to the frequency parameters estimated in AMUD 1.0, the modified version also estimates a frequency track, using a Viterbi-algorithm [3] minimizing the distance of the frequency points. Here we only consider those segments where the criterion $m1 \geq \Theta_{off}$ $\Delta := m1 - (m2 + m3) \geq \phi_{off}$ is fulfilled. From the values of the frequency tracks on those valid segments we also calculate the following parameters:

Mean frequency	The average of the detected frequencies in the track
USV length	The length of the detected segment
Frequency slope	A linear regression was applied on the valid points in the track resulting in an approximation representation $y = \alpha x + d$. The slope α is used.
Mean amplitude	The average of the amplitudes of the points on the track.

In pilot tests we found out that those values are reliable parameters for a classification of USVs, while noting that they are not always easily interpretable (e.g. the slopes for USVs with jumps).

An additional quality criterion for the segmentation is also calculated, allowing a statistical tradeoff for true positives. For each detected segment with K time points (‘frames’), i.e. K spectra, the number $k_1$where $m1 \geq \Theta_{on}$ and k_2 where $\Delta := m1 - (m2 + m3) \geq \phi_{on}$ are used. We consider $Xc = [\,cor(m_1, \Delta)]_+$, i.e. the correlation of m_1 and Δ, setting all negative values to zero. (Note that all considered variables are vectors of length K.) Let $Xo = \frac{k_1 \cdot k_2}{K^2}$, which is a combination of the ration of the frames of the given segment where the conditions in 2’) are fulfilled.

From a randomly chosen test set of 3960 segments we have collected the distribution of true positives in relation to those two variables (Fig S1). This map, scaled to the range 0 to 100 using a discretization of the interval [0,1] into 10 values, was used to create the quality criterion for the algorithm.

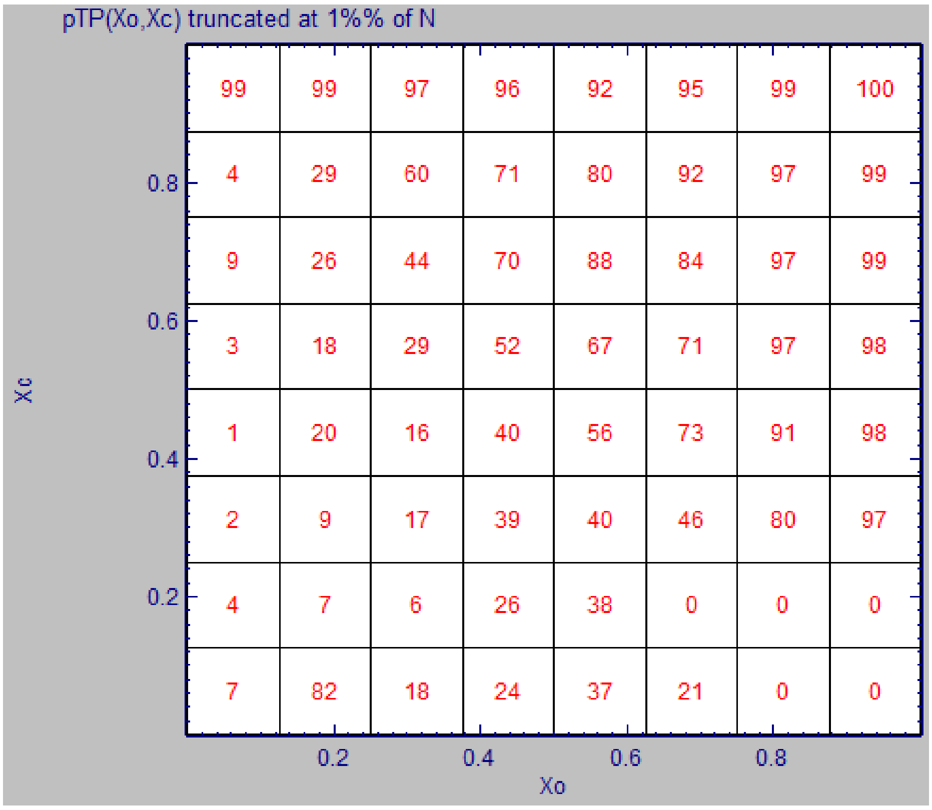

S1 Fig. Distribution of true positives depending on $\boldsymbol{Xc}$ and $\boldsymbol{Xo}$.

For a given segment, a quality value ρ between 0 and 1 is used as classification threshold by comparing values Xc and Xo and the wanted value ρ (multiplier by 100) in the empirical table (in Fig S1), ignoring the last two lines, as they contain some statistical outliers and are in the low performance area. This approach was introduced to improve detection of more true positives, which was an issue with AMUD 1.0, especially for noisy data. The new criterion performed better by ignoring some background sounds, as well as being able to detect softer, shorter USVs. Increasing the number of true positives was one of the goals of the new method. This tool is free for scientific (non-profit) use and available here: https://www.kfs.oeaw.ac.at/doc/amud/AMUD1b.sts (Script);
Readme: https://www.kfs.oeaw.ac.at/doc/amud/AMUD1b-Readme.odt.

Evaluation of USV detection performance (A-MUD 3.2)

We evaluated the error rates of the new version of our automatic detection tool, including the true positives (TP), true negatives (TN), false positive (FP), and false negatives (FN), using the default settings to detect USVs in N=9693 segments in 14 recordings that were used to develop and validate A-MUD 1.0 [1]. In Fig S2 we plot sensitivity (TPR, green line) and specificity (TNR, red line) versus ρ (=QP in the figures). Sensitivity is defined by $TPR = \frac{TP}{TP+FN}$, whereas specifity is $TNR = \frac{TN}{TN+FP}$ (see e.g [4]). So for ρ between 0.1 and 0.9 both values were above 0.8. The graph suggests using values for ρ between 0.4 and 0.8.

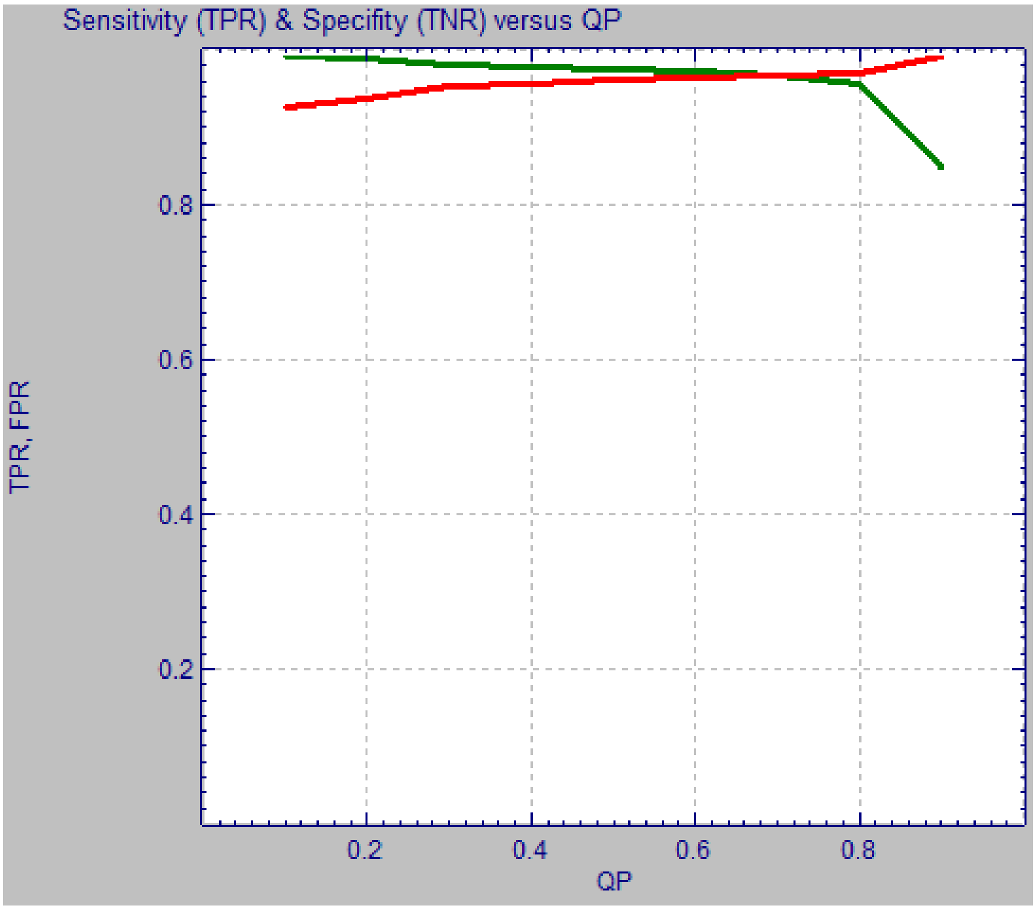

S2 Fig. Sensitivity (green) and specifity (red) depending on ρ.

In Fig S3 we plot accuracy versus the segment quality value ρ. Here accuracy is defined by $c = \frac{TP+TN}{N} = \frac{TP+TN}{TP+TN+FP+FN}$. Again, for non-extreme values of ρ, i.e. between 0.1 and 0.9, the accuracy is above 0.9.

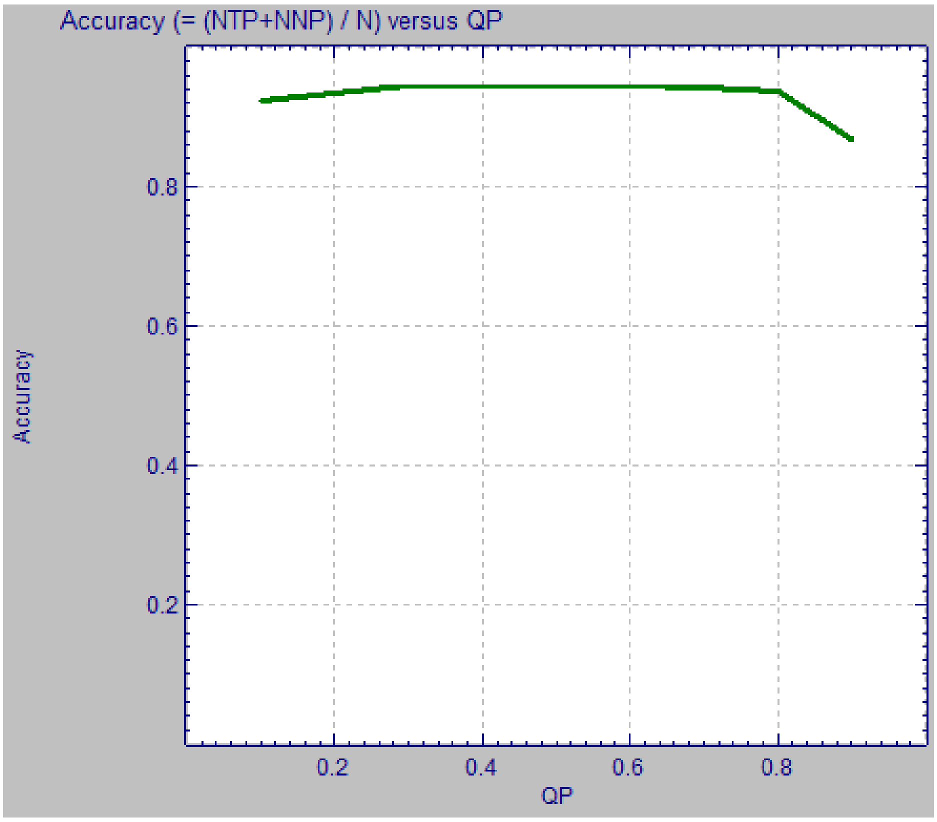

S3 Fig. Accuracy depending on quality value.

A well-established method to judge the performance of a classification model is the receiver operating characteristic (ROC) curve, which plots the true positive rate (TPR) against the false positive rate (FPR). For AMUD 3.2 this is plotted in Fig S4 for ρ=0.5 against the purely random decision (the diagonal). The area under the ROC curve (AUC) is one value for the quality of the method, which was 0.989, and values > 0.9 can be considered to be excellent. The Gini coefficient $G1 = 2 * AUC - 1 = 0.978$ is a measure used for statistical dispersion. The maximal Youden's index $Jmax = TPR + TNR - 1 = 0.937$ generally is considered a measure for the 'informedness' of a classification (the largest distance to the diagonal, see the red dot). All the values show that the performance of AMUD 3.2 was outstanding for our dataset.

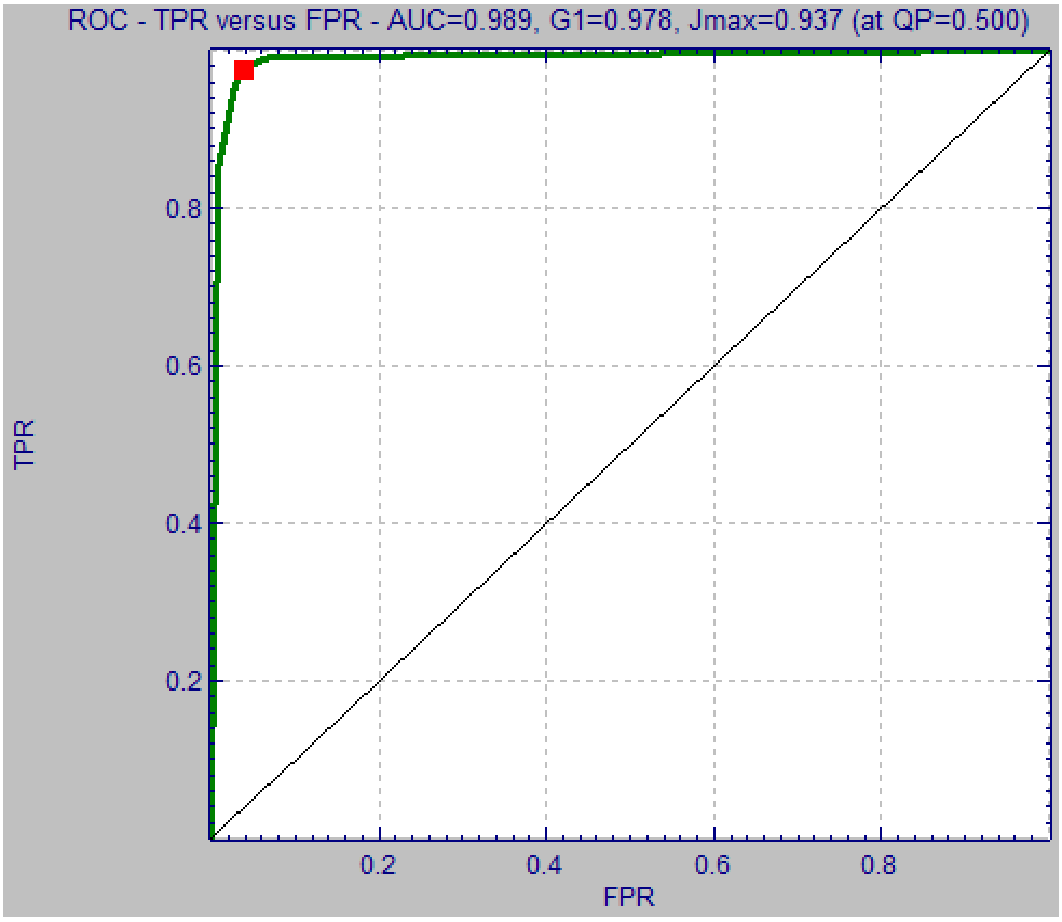

S4 Fig. ROC curve, true positives (TPR) versus false positives (FPR).

S1 Results

Vocal repertoire of USVs (repertoire diversity and composition) omitting unclassified USVs

GLM analysis showed that all models for repertoire diversity had the same ranking of AICcs (294.6, 295.7, 300.7) for timepoint, treatment, and null models. Compared with controls, repertoire diversity was significantly elevated on days 1 ($p<0.001$), 20 ($p=0.029$), and 30 ($p=0.029$, Fig S5).

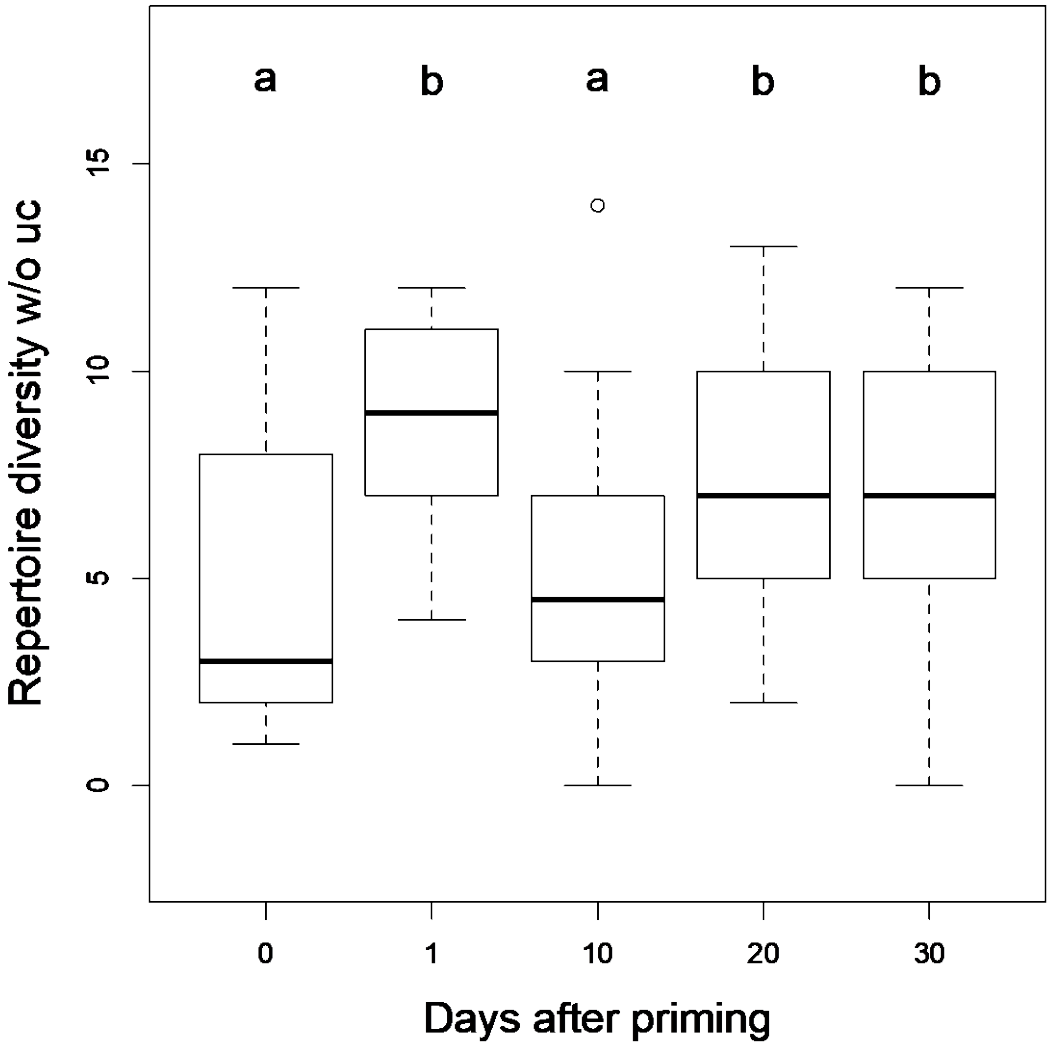

S5 Fig. Repertoire diversity of USV with or without priming. Boxplots with medians of repertoire diversity of unprimed (0) and primed males (≥1). Different letters denote significant differences ($p<0.05$).

We examined priming effects in repertoire composition, i.e., vocalization type occurrence per group, using the same two statistical non-parametric multivariate approaches as described in the main text. Both analyses showed that groups differed and that the unprimed

males had the most distinctive vocal repertoire composition (ANOSIM: R=0.091, p=0.039) and (PERMANOVA: F(4,43)=1.697, p=0.039). We visualized the data using non-metric multi-dimensional clustering (nMDS), and plotted the occurrence of each vocalization type per experimental group. The different vocalization types (represented by letters) are positioned according to their highest clustering. The main difference appears to be due to unprimed males emitting more short (s) and less complex vocalizations than the other mice, whereas the 1d primed males have more ultra-high (uh), complex (e.g. c2, c3, c4) and less short (s) and ultra-short (us) vocalizations than the other mice (Fig S6). Further visualization of the repertoire composition is shown in pie charts (Fig S7).

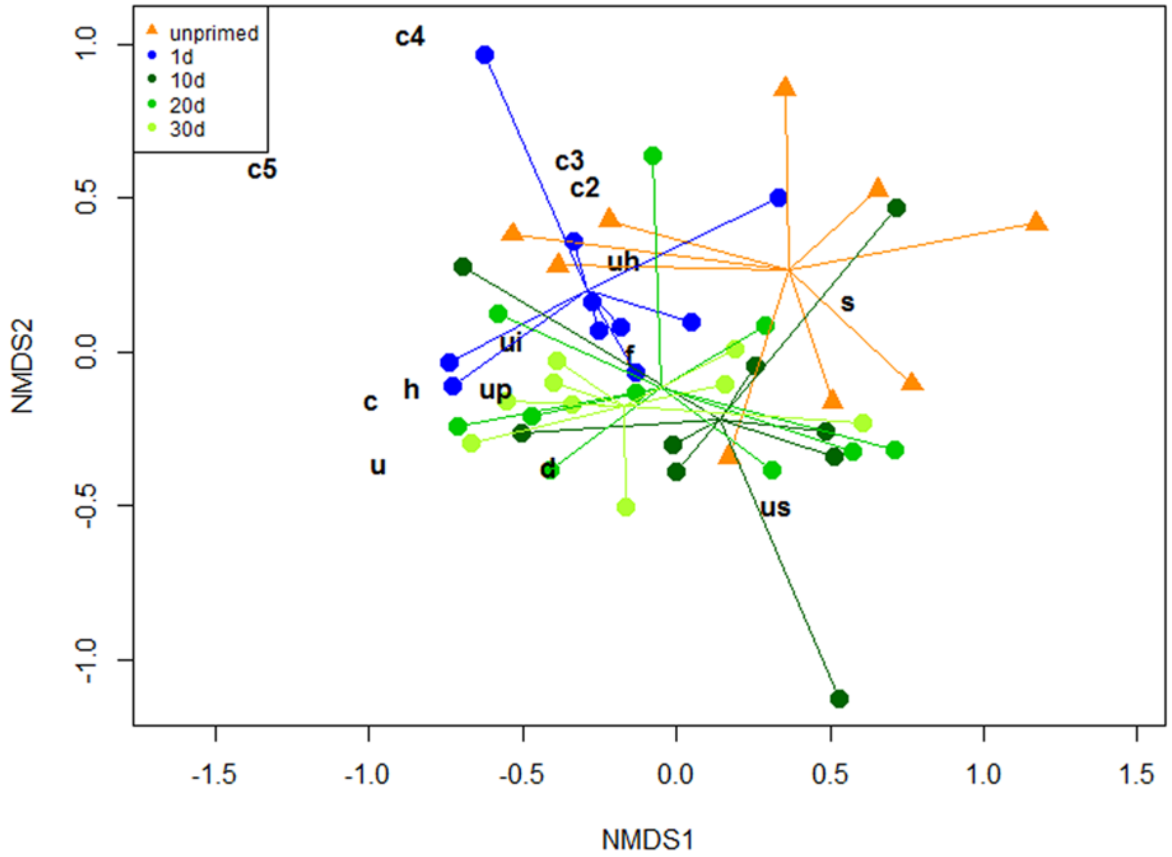

S6 Fig. Non-metric multi-dimensional clustering of USV vocalization type occurrence according to priming groups (nMDS: stress=0.16). Mice are clustered according to the amount of each vocalization type emitted, omitting unclassified USVs (see also Table 1 for abbreviations of USV types). Mice are group-color coded: unprimed males in orange triangles, 1d primed males in blue circles, 10d primed males in dark green circles, 20d primed males in green circles and 30d primed males in light green circles.

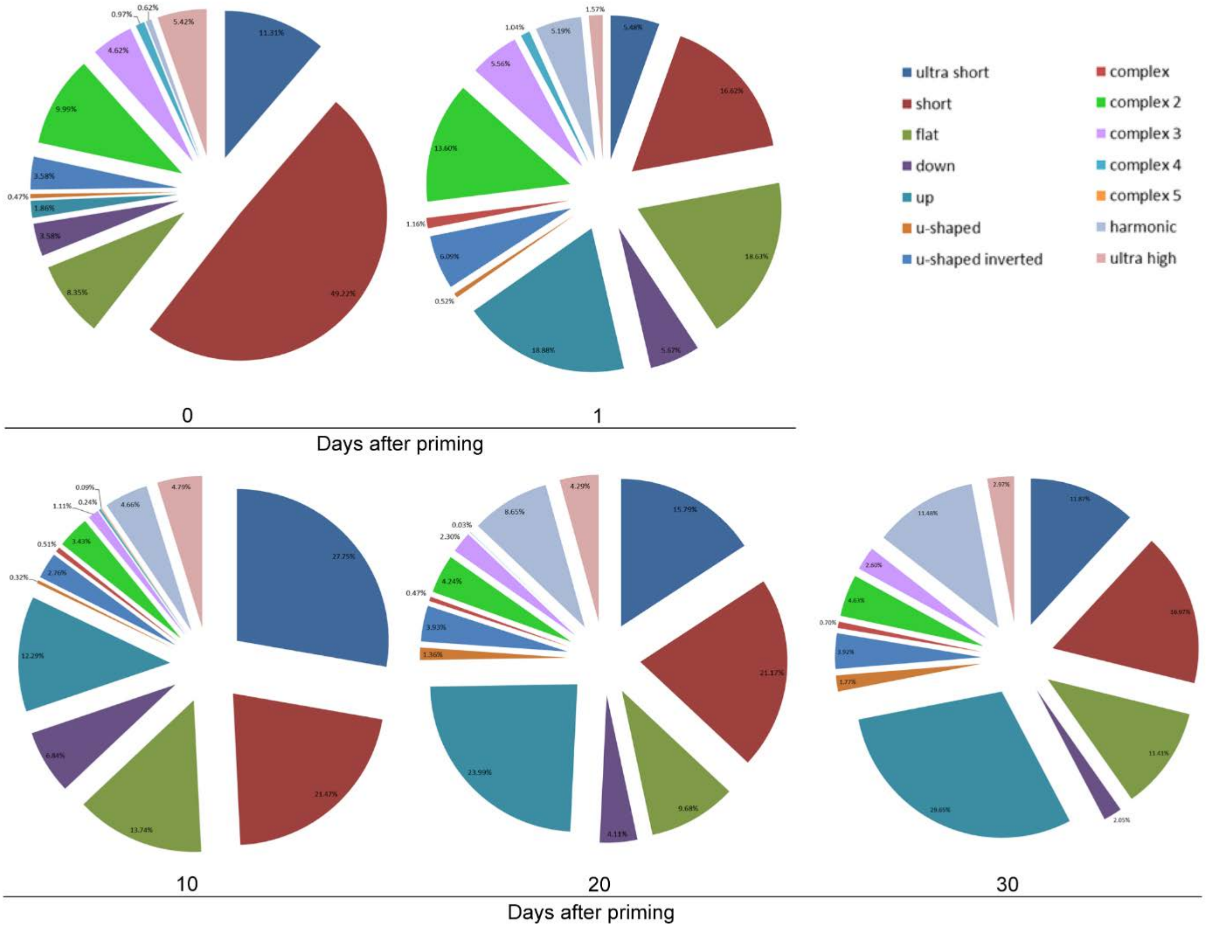

S7 Fig. Proportions of the different types of vocalizations emitted by mice in the treatment and control groups. Pie charts show the mean proportions (%) of the occurrence of vocalization types emitted, omitting unclassified USVs. The legend shows the vocalization types.

References

1. Zala SM, Reitschmidt D, Noll A, Balazs P, Penn DJ. Automatic mouse ultrasound detector (A-MUD): A new tool for processing rodent vocalizations. PLoS ONE. 2017;12(7):e0181200. doi: 10.1371/journal.pone.0181200.

2. Oppenheim AV, Schafer RW. Digital Signal Processing: Pearson; 2015.

3. Forney Jr. GD. The Viterbi algorithm. Proceedings of the IEEE. 1973;61(3):268-78. doi: 10.1109/PROC.1973.9030.

4. Macmillan NA, Creelman DC. Detection Theory: A User's Guide. 2nd ed: Lawrence Erlbaum; 2004. 512 p.
